# Cilia interactome with predicted protein–protein interactions reveals connections to Alzheimer’s disease, aging and other neuropsychiatric processes

**DOI:** 10.1038/s41598-020-72024-4

**Published:** 2020-09-24

**Authors:** Kalyani B. Karunakaran, Srilakshmi Chaparala, Cecilia W. Lo, Madhavi K. Ganapathiraju

**Affiliations:** 1 Supercomputer Education and Research Centre, Indian Institute of Science, Bangalore, India; 2 Department of Biomedical Informatics, University of Pittsburgh, Pittsburgh, PA USA; 3 Health Sciences Library System, University of Pittsburgh, Pittsburgh, PA USA; 4 Department of Developmental Biology, School of Medicine, University of Pittsburgh, Pittsburgh, PA USA; 5 Intelligent Systems Program, School of Computing and Information, University of Pittsburgh, Pittsburgh, PA USA

**Keywords:** Developmental biology, Molecular neuroscience

## Abstract

Cilia are dynamic microtubule-based organelles present on the surface of many eukaryotic cell types and can be motile or non-motile primary cilia. Cilia defects underlie a growing list of human disorders, collectively called ciliopathies, with overlapping phenotypes such as developmental delays and cognitive and memory deficits. Consistent with this, cilia play an important role in brain development, particularly in neurogenesis and neuronal migration. These findings suggest that a deeper systems-level understanding of how ciliary proteins function together may provide new mechanistic insights into the molecular etiologies of nervous system defects. Towards this end, we performed a protein–protein interaction (PPI) network analysis of known intraflagellar transport, BBSome, transition zone, ciliary membrane and motile cilia proteins. Known PPIs of ciliary proteins were assembled from online databases. Novel PPIs were predicted for each ciliary protein using a computational method we developed, called High-precision PPI Prediction (HiPPIP) model. The resulting cilia “interactome” consists of 165 ciliary proteins, 1,011 known PPIs, and 765 novel PPIs. The cilia interactome revealed interconnections between ciliary proteins, and their relation to several pathways related to neuropsychiatric processes, and to drug targets. Approximately 184 genes in the cilia interactome are targeted by 548 currently approved drugs, of which 103 are used to treat various diseases of nervous system origin. Taken together, the cilia interactome presented here provides novel insights into the relationship between ciliary protein dysfunction and neuropsychiatric disorders, for e.g. interconnections of Alzheimer’s disease, aging and cilia genes. These results provide the framework for the rational design of new therapeutic agents for treatment of ciliopathies and neuropsychiatric disorders.

## Introduction

Cilia are dynamic organelles projecting from the surface of many types of eukaryotic cells. They detect changes in the extracellular environment and transduce signals into the cell to regulate a wide variety of physiological and developmental processes. They can be either motile or non-motile, and exhibit a microtubule organization of 9 + 2 or 9 + 0, respectively^1^. Primary cilia are sensory organelles modulating several core signaling and cellular polarity pathways that are fundamental for tissue homeostasis and embryonic development^2^. Motile cilia drive the flow of bodily fluids including mucus and cerebrospinal fluid^3,4^. Defects involving the primary cilia are observed in various human ciliopathies such as Bardet-Biedl syndrome (BBS), Joubert syndrome and Meckel–Gruber syndrome. Motile cilia defects are seen in primary ciliary dyskinesia (PCD), male infertility and laterality defects^5^.

The cilium is a complex organelle comprising over 600 proteins^1^. Underscoring their functional importance, many of these ciliary proteins are highly evolutionarily conserved including the intraflagellar transport (IFT) complexes located within the axoneme involved in bidirectional protein transport between the ciliary base and the tip, complexes localizing to the transition zone (TZ) at the ciliary base acting as a ‘ciliary gate’ regulating protein trafficking into and out of the cilia and BBSomes mediating cilia assembly^6–8^.

Primary cilium is increasingly viewed as a hub for neuronal signalling. A large body of evidence has emerged demonstrating the role of cilia in the development and function of the central nervous system (CNS)^9–11^. Gene knockdown of BBS proteins such as BBS1, BBS4-5, BBS7, and BBS9-12 lead to cortical defects and improper neuronal migration, highlighting the significance of cilia genes in brain development^12^. Additionally, neural tube defects are observed in the brain with the disruption of cilia-transduced sonic hedgehog signaling (Shh) and Wnt signaling^13,14^. Indeed, many ciliopathies are known to be associated with neurological deficits such as developmental delays, cognitive impairment and neuropsychiatric disorders including ataxia, autism spectrum disorders and schizophrenia^10,12^. Importantly, the ciliary proteins AHI1, ARL13b, CDKL5 and EFHC1 have been implicated in autism spectrum disorder, epilepsy, and schizophrenia^15–18^. A recent study identified neuropsychiatric risk genes (NEK4, SDCCAG8, FEZ1, CEP63, PDE4B and SYNE1) to be linked to cilia assembly and function^15^. In addition, several ciliary proteins interact with proteins that are known to play a role in neuropsychiatric disorders: PCM1, BBS4 with DISC1 in schizophrenia, bipolar disorder and depression^19,20^, KIF3A, PCNT with DCDC2 in dyslexia^21,22^, and PCM, AHI1 with HTT in Huntington disease^12,23,24^. Hydrocephalus, a phenotype observed frequently in BBS and other ciliopathies, may reflect the role of motile cilia in the flow of cerebrospinal fluid in the brain^10^. Ciliopathies have also been associated with obesity, suggesting a role for cilia in the neural circuitry responsible for monitoring food intake and satiety^25^. The obesity-related genes MC4R and ADCY3 co-localize to primary cilia of hypothalamic neurons, and impairing this localization or blocking their signalling in primary cilia led to gain in body weight in mice^26^.

Given the importance of large multi-protein complexes in its assembly and function, knowledge of the protein–protein interactions (PPIs) of ciliary proteins would help to elucidate the potential role of cilia biology in neuropsychiatric diseases. Studies based on PPI networks have significantly advanced our knowledge of specific proteins or the diseases that they are associated with, such as DISC1 in schizophrenia, or the NPHP-JBTS-MKS protein complex in ciliopathies^27^. DISC1 was a novel protein with well-characterized domains but of unknown function with no known human homolog, when it was identified as being associated with schizophrenia^28,29^. To understand the function of DISC1, its PPIs were determined using yeast 2-hybrid technology^30,31^. This led to a large number of studies, which connected DISC1 to cAMP signaling, axon elongation and neuronal migration. A study revealed that the role played by DISC1 in dopamine signaling, which is implicated in schizophrenia, may also involve primary cilia on neurons^19^. DISC1 localized to primary cilia on rat striatal neurons and was found to be involved in the formation and maintenance of cilia with certain dopamine receptors^19^. The PPI network of ciliary proteins CEP290 and RPGR revealed their connection to photoreceptors, and disruption of this network has been shown to cause blindness on rapid degeneration of photoreceptors, a finding associated with several ciliopathies^32^.

Large-scale proteomic and protein interactome analyses have significantly advanced our understanding of its role in developmental biology and disease^33–37^. Multidimensional protein identification technology (MudPIT) was used to identify 195 candidate primary cilia proteins localizing to sensory cilia, or linked to known ciliopathies^33^. 850 interactors of nine NPHP/JBTS/MKS proteins (i.e. Nephronophthisis/Joubert/Meckel-Gruber syndromes) were identified using the G-LAP-Flp purification strategy, and several cilia-specific modules, namely ‘NPHP1-4-8’ functioning at the apical surface, ‘NPHP5-6’ at centrosomes and ‘MKS’ linked to hedgehog signaling were uncovered^34^. In another study, in vivo proximity-dependent biotinylation (BioID) was used to identify more than 7,000 interactions of 58 centriole, satellite and ciliary transition zone proteins, which revealed protein modules involved in cilia and centrosome biogenesis^35^. The interactome of CPLANE (ciliogenesis and planar polarity effector) proteins, namely that of *Inturned* (INTU), *Fuzzy* (FUZ) and *Wdpcp* (WDPCP), consisting of ~ 250 interactions, was identified using LAP-tagged immunoprecipitation, and it was shown that the CPLANE proteins govern IFT-A/B trafficking^36^. Systematic tandem affinity purifications coupled to mass spectrometry was employed to identify 4,905 interactions and 52 complexes for 217 proteins with known or suspected involvement in ciliary function or disease, and this study linked vesicle transport, the cytoskeleton and ubiquitination to ciliary signaling and proteostasis^37^. None of these experimental methods are single handedly capable of identifying all the possible interactions of ciliary genes. In fact, it is the ability of an experimental method to discover interactions not detected by another method that makes it truly valuable. Machine learning methods can computationally predict new interactions that other high throughput detection methods may fail to capture and serve as hypotheses-generation methods that may be validated by other experimental methods. Here, we applied computational method that we developed previously to discover novel PPIs of 165 ciliary proteins and analyzed the resulting ciliary PPI interactome for novel associations and potential connections to neuropsychiatric diseases.

## Experimental procedures

### Dataset

Compilation of Cilia Gene List: We obtained a list of 165 cilia genes that were curated from literature by prioritizing the genes based on their association with cilia from Dr. Gregory Pazour’s lab building upon their prior work^38^. This list includes IFT proteins, BBS proteins, TZ proteins, ciliary membrane proteins, and proteins restricted to motile cilia. Known PPIs were collected from Human Protein Reference Database (HPRD)^39^ and Biological General Repository for Interaction Datasets (BioGRID)^40^. Gene-drug associations and ATC classifications were collected from DrugBank^41^, while neuropsychiatric gene-disease associations were collected from the GWAS catalog (www.ebi.ac.uk/gwas/). Random gene sets used in shortest path comparisons were sampled from about twenty thousand human proteins listed in the Ensembl database (www.ensembl.org).

Novel PPIs were predicted using the HiPPIP model that we developed^42^. Each ciliary protein (say C_1_) was paired with each of the other human genes say, (G_1_, G_2_, … G_n_), and each pair was evaluated with the HiPPIP model. The predicted interactions of each of the cilia genes were extracted, which resulted in 620 newly discovered PPIs of cilia genes. The average shortest path distance was computed using the Networkx package in python. Pathway associations were computed using Ingenuity Pathway Analysis suite. GO term enrichment was carried out using BinGO^43^; for each C_1_, a list of its known and predicted interacting partners (i.e. B_1_, B_2_, … B_*n*_) are given as input to BinGO, which extracts the GO terms of all these genes and finds which of the GO terms are statistically enriched in comparison to the background distribution of GO terms of all human proteins. All statistically significant terms are assigned as network-based enriched GO terms of C_1_.

Gene expression datasets in Gene Expression Omnibus were used to compute the overlap of the cilia interactome with genes differentially expressed in various neuropsychiatric disorders: major depressive disorder (GSE53987^44^), schizophrenia (GSE17612^45^), bipolar disorder (GSE12679^46^), autism spectrum disorder (GSE18123^47^), Alzheimer’s disease (GSE29378^48^ and GSE28146^49^), Parkinson’s disease (GSE28894) and non-syndromic intellectual disability (GSE39326^50^). Genes with fold change > 2 or < ½ were considered as significantly overexpressed and underexpressed respectively at *p* value < 0.05. A gene with transcripts per million ≥ 2 was considered to be ‘expressed’ while analyzing the overlap of the interactome with genes expressed in the amygdala, anterior cingulate cortex, caudate, cerebellum, frontal cortex, hippocampus, hypothalamus, nucleus accumbens, putamen, spinal cord and substantia nigra extracted from GTEx^51^. Time-dependent gene expression variation in the hippocampal region was extracted from BrainSpan Atlas containing RNA-Seq data from post-conceptional weeks to middle adulthood^52^. 78 genes associated with Alzheimer’s disease were extracted from DisGeNET^53^ (with score > 0.2 to include only expert-curated disease-gene associations). Then, to construct the Alzheimer’s disease interactome, whose overlap was to be checked with the cilia interactome, 4,742 known PPIs extracted from HPRD^54^ and BioGRID^55^, and 490 computationally predicted PPIs of these 78 genes were assembled. The biological validity of the interactome was shown by the fact that 676 genes out of the 3,944 genes in the AD interactome are differentially expressed in CA1 hippocampal gray matter from patients with severe Alzheimer's disease versus healthy controls (GSE28146^49^), out of which 71 were novel interactors (*p* value = 1.138e−20).

## Results

**Figure 1:**
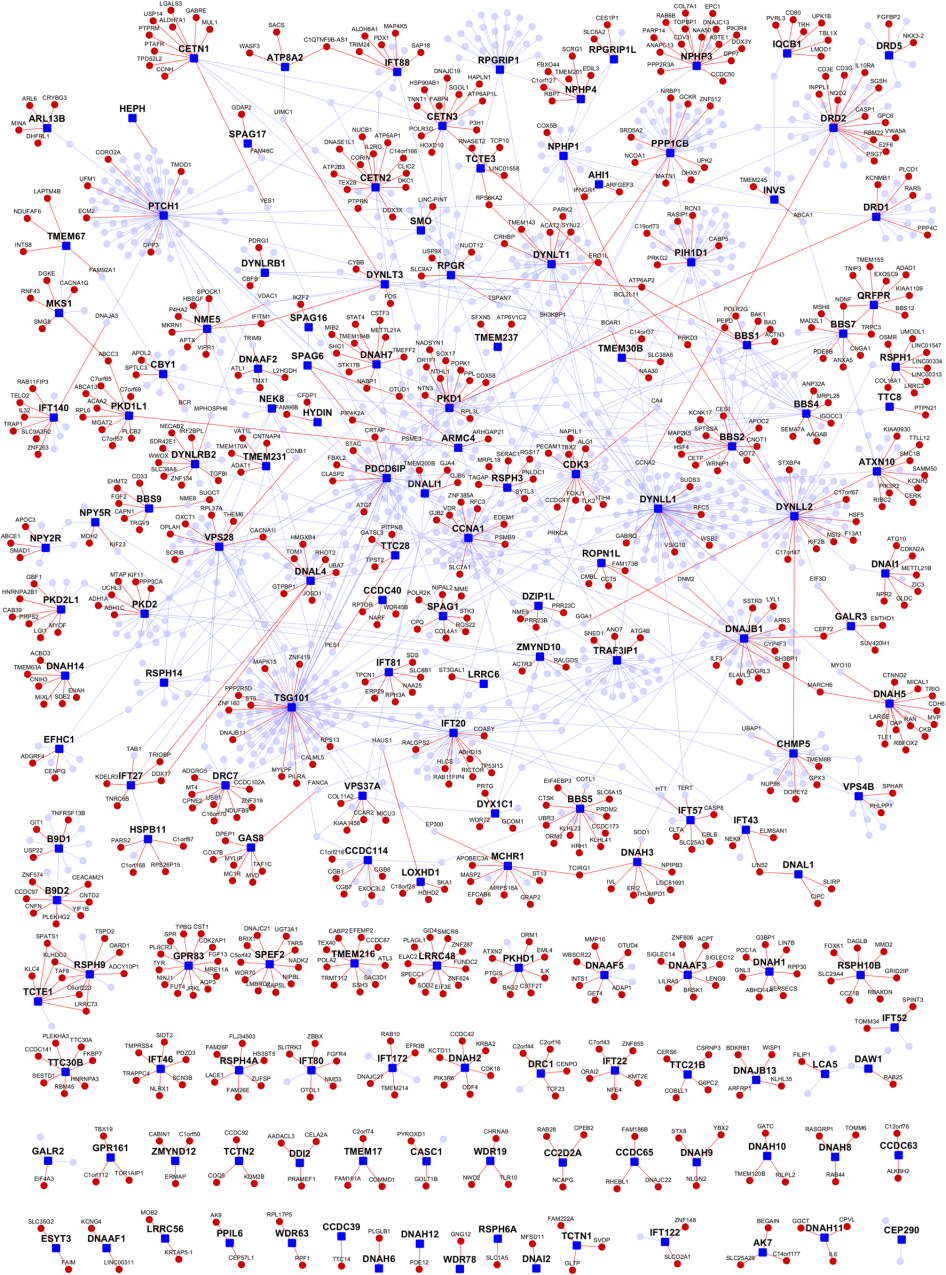
Cilia interactome. Cilia genes are shown as small dark-blue colored nodes and interactor genes are larger round nodes; the interactors are colored in light blue if they are previously known interactors and in red if they are found only through novel PPIs. PPIs are shown as edges, where blue color edges are known PPIs and red color edges are novel predicted PPIs. Most genes at the bottom of the figure have had zero known PPIs, and have multiple novel predicted PPIs.

We assembled a list of 165 genes encoding proteins known to be associated with primary and/or motile cilia, including IFT, BBS, TZ, and ciliary membrane proteins, as well as proteins restricted to motile cilia. Known PPIs of ciliary proteins were assembled from HPRD and BioGRID^40,56^. Novel PPIs were predicted for each of the cilia genes using our High-precision Protein–Protein Interaction Prediction (HiPPIP) model^42^. In this manner, a ciliary protein interactome was assembled comprising 165 ciliary proteins (red color square shaped nodes) with 1,011 known PPIs (blue edges) and 765 novel PPIs (red edges) that connect to 800 previously known interactors (light blue nodes) and 705 novel interactors (red nodes) (Fig. 1 and Table 1). We predicted 216 new interactions for 50 out of the 56 cilia genes that had no known PPIs. For example, GPR83 has 12 novel PPIs, LRRC48 has 10, PKD1L1 has 10, and SPEF has 10 novel PPIs. The number of known and novel PPIs of cilia genes are given in Supplementary File 1, and the lists of all genes and PPIs is given in Supplementary File 2.

**Table 1:** Novel interactors of each of the cilia genes.

Cilia gene	K	N	Novel interactors
AHI1	1	2	ARFGEF3, IFNGR1
AK7	0	3	BEGAIN, C14orf177, SLC25A29
ARL13B	1	4	MINA, CRYBG3, ARL6, DHFRL1
ARMC4	3	3	ARHGAP21, OTUD1, PIP4K2A
ATP8A2	1	3	C1QTNF9B-AS1, WASF3, SACS
ATXN10	9	9	TTLL12, RIBC2, SMC1B, GGA1, KCNH3, PIK3R2, KIAA0930, SAMM50, CERK
B9D1	7	3	TNFRSF13B, USP22, GIT1
B9D2	2	7	PLEKHG2, YIF1B, CCDC97, CNFN, CEACAM21, ZNF574, CNTD2
BBIP1	0	0	None
BBS1	13	6	ACTN3, BAK1, BAD, ATP6AP2, PEPD, POLR2G
BBS2	11	10	APOC2, WRNIP1, CETP, GOT2, SPTSSA, CES1, CNOT1, HSF4, MAP2K5, KCNK17
BBS4	13	5	ANP32A, AAGAB, MRPL28, SEMA7A, IGDCC3
BBS5	5	11	SLC6A15, CCDC173, KLHL41, CTSK, COTL1, EIF4EBP3, UBR3, KLHL23, ORM2, PRDM2, HRH1
BBS7	5	8	ANXA5, PDE8B, MSH6, MAD2L1, TRPC3, NDNF, QRFPR, CNGA1
BBS9	0	6	NME8, CD33, EHMT2, TRGV9, FGF2, CAPN1
CASC1	1	2	GOLT1B, PYROXD1
CBY1	8	3	APOL2, BCR, SPTLC3
CC2D2A	0	3	CPEB2, NCAPG, RAB28
CCDC114	8	4	CGB8, EXOC3L2, CGB7, CGB1
CCDC135	6	0	None
CCDC39	0	1	TTC14
CCDC40	1	3	NARF, RPTOR, WDR45B
CCDC63	0	2	ALKBH2, C12orf76
CCDC65	0	3	FAM186B, DNAJC22, RHEBL1
CCNA1	32	7	VDR, RFC3, GJB2, ZNF385A, PSMB9, SLC7A1, EDEM1
CDK3	17	10	CCDC47, CA4, ALG1, TLK2, ITIH4, PRKCA, PECAM1, NAP1L1, FOXJ1, TBX2
CEP290	2	0	None
CETN1	4	10	ALDH7A1, CCNH, GABRE, MUL1, YES1, LGALS3, PTPRM, USP14, TPD52L2, PTAFR
CETN2	9	12	ATP6AP1, C14orf166, ATP2B3, NUCB1, DKC1, PTPRN, CLIC2, DNASE1L1, TEX28, IL2RG, DDX3X, CORIN
CETN3	6	11	ATP6AP1L, HSP90AB1, HOXD10, SGOL1, FABP4, HAPLN1, TNNT1, P3H1, DNAJC19, POLR3G, PRKD3
CHMP5	14	6	NUP98, UBAP1, DYNLL2, TMEM8B, DOPEY2, GPX3
DAW1	2	1	RAB25
DDI2	0	3	AADACL3, CELA2A, PRAMEF1
DNAAF1	0	2	LINC00311, KCNG4
DNAAF2	1	4	ATL1, TRIM9, L2HGDH, TMX1
DNAAF3	0	7	ACPT, BRSK1, SIGLEC14, SIGLEC12, LENG9, LILRA5, ZNF606
DNAH1	0	7	ABHD14A, SEPSECS, LIN7B, POC1A, RPP30, GNL3, G3BP1
DNAH10	0	3	GATC, RILPL2, TMEM120B
DNAH11	0	3	CPVL, GGCT, IL6
DNAH12	0	1	PDE12
DNAH14	1	6	ACBD3, CNIH3, ENAH, TMEM63A, SDE2, MIXL1
DNAH17	0	0	None
DNAH2	0	6	CDK18, CCDC42, KCTD11, ODF4, PIK3R6, KRBA2
DNAH3	0	7	ERI2, TCIRG1, SOD1, THUMPD1, NPIPB3, LOC81691, IVL
DNAH5	0	13	CTNND2, CKB, CDH6, DAP, LARGE, MYO10, TLE1, MVP, RAN, TRIO, MARCH6, MICAL1, RBFOX2
DNAH6	0	1	PLGLB1
DNAH7	2	9	CSTF3, SHC1, STK17B, STAT4, METTL21A, TMEFF2, NABP1, TMEM194B, MIB2
DNAH8	0	3	TOMM6, RAB44, RASGRP1
DNAH9	0	3	YBX2, NLGN2, STX8
DNAI1	2	6	ATG10, CDKN2A, ZIC3, NPR2, METTL21B, GLDC
DNAI2	0	1	MFSD11
DNAJB1	28	10	CYP4F3, ARR3, CEP72, ADGRL3, ELAVL3, DNM2, ILF3, LYL1, SH3BP1, SSTR2
DNAJB13	0	4	BDKRB1, ARFRP1, KLHL35, WISP1
DNAL1	0	3	CIPC, LIN52, SLIRP
DNAL4	9	9	DDX17, CACNA1I, UBA7, TRIOBP, TOM1, GTPBP1, RHOT2, JOSD1, HMGXB4
DNALI1	5	3	GJA4, GJB5, TMEM200B
DRC1	1	4	C2orf44, CENPO, C2orf16, TCF23
DRC7	6	8	CCDC102A, ADGRG5, CPNE2, C16orf70, ZNF319, USB1, NDUFB9, MT4
DRD1	11	5	AP3M1, PLCD1, RARS, PPP4C, KCNMB1
DRD2	20	13	CASP1, CCNA2, CD3G, CD3E, GPC6, SGSH, VWA5A, INPPL1, IL10RA, PSG7, NQO2, RBM22, E2F6
DRD5	5	2	NKX3-2, FGFBP2
DYNLL1	76	5	RFC5, SUDS3, GABRQ, WSB2, VSIG10
DYNLL2	47	9	C17orf67, CHMP5, C17orf47, HSF5, STXBP4, F13A1, KIF2B, PRKD3, MSI2
DYNLRB1	6	2	CBFB, PDRG1
DYNLRB2	4	8	WWOX, SDR42E1, TGFBI, ZNF134, SLC38A8, NECAB2, IRF2BPL, MPHOSPH6
DYNLT1	28	7	CRHBP, ACAT2, SYNJ2, RPS6KA2, TMEM143, PARK2, ERO1L
DYNLT3	7	4	CYBB, IFITM1, UIMC1, FOS
DYX1C1	4	3	GCOM1, PRTG, WDR72
DZIP1L	4	3	PRR23B, NME9, PRR23C
EFHC1	6	2	CENPQ, ADGRF4
ESYT3	0	2	FAIM, SLC35G2
GALR2	1	1	EIF4A3
GALR3	0	4	EIF3D, CEP72, ENTHD1, SUV420H1
GAS8	0	7	FANCA, COX7B, DPEP1, MYLIP, MVD, MC1R, TAF1C
GPR161	0	3	C1orf112, TOR1AIP1, TBX19
GPR83	0	12	FUT4, CST1, CDK2AP1, AQP3, FGF13, PLSCR3, TYR, TPBG, JRKL, MRE11A, NINJ1, SPR
HEPH	0	1	MSN
HSPB11	2	4	C1orf168, C1orf87, RPS26P15, PARS2
HYDIN	1	2	FAM96B, CFDP1
IFT122	1	2	ZNF148, SLCO2A1
IFT140	4	7	DNAJA3, ZNF263, SLC9A3R2, RAB11FIP3, TELO2, IL32, TRAP1
IFT172	2	4	EFR3B, DNAJC27, RAB10, TMEM214
IFT20	28	7	ABHD15, COASY, HLCS, RALGPS2, RICTOR, TP53I13, RAB11FIP4
IFT22	0	5	C7orf43, NFE4, ORAI2, KMT2E, ZNF655
IFT27	2	5	DDX17, TNRC6B, KDELR3, TRIOBP, TAB1
IFT43	2	3	ELMSAN1, LIN52, NEK9
IFT46	0	6	NLRX1, TMPRSS4, SCN3B, TRAPPC4, SIDT2, PDZD3
IFT52	1	2	SPINT3, TOMM34
IFT57	4	6	CLTA, CASP8, CBLB, HTT, SLC25A3, TERT
IFT74	0	0	None
IFT80	1	5	FGFR4, OTOL1, SLITRK3, NMD3, ZBBX
IFT81	1	6	ERP29, NAA25, TPCN1, SLC8B1, SDS, RPH3A
IFT88	6	6	C1QTNF9B-AS1, ALDH8A1, MAP4K5, PDX1, TRIM24, SAP18
INVS	5	2	ABCA1, TMEM245
IQCB1	1	6	CD80, UPK1B, TRH, LMOD1, PVRL3, TBL1X
LCA5	3	1	FILIP1
LOXHD1	0	4	HDHD2, HAUS1, C18orf25, SKA1
LRRC48	0	10	ELAC2, GID4, EIF3E, FUNDC2, SMCR8, ZNF287, SPECC1, PLAGL1, ZNF624, SOD2
LRRC56	0	2	KRTAP5-1, MOB2
LRRC6	2	1	ST3GAL1
MCHR1	2	8	GRAP2, EP300, APOBEC3A, MASP2, EFCAB6, TCIRG1, MRPS18A, ST13
MKS1	2	5	ABCC3, DGKE, CACNA1G, RNF43, SMG8
NEK8	4	0	None
NME5	2	8	HBEGF, APTX, MKRN1, IFITM1, VDAC1, SPOCK1, VIPR1, P4HA2
NME8	1	2	BBS9, SUGCT
NPHP1	15	2	BCL2L11, COX5B
NPHP3	2	15	CCDC50, ANAPC13, COL7A1, ASTE1, NAA50, DPP7, EPC1, DDX3Y, CDV3, DNAJC13, PIK3R4, TOPBP1, PARP14, PPP2R3A, RAB6B
NPHP4	1	6	FBXO44, EDIL3, C1orf127, TMEM201, SCRG1, RBP7
NPY2R	4	3	APOC3, ABCE1, SMAD1
NPY5R	4	2	MDH2, KIF23
PDCD6IP	37	5	ATG7, CLASP2, FBXL2, CRTAP, STAC
PIH1D1	31	5	CABP5, C19orf73, RCN3, RASIP1, PRKG2
PKD1	24	9	NTHL1, NADSYN1, PDPK1, OR1F1, DDX58, NTN3, RPL3L, SOX17, PPL
PKD1L1	0	10	C7orf69, ABCC3, C7orf65, ACAA2, C7orf57, MGAT2, ABCA13, RPL6, PLCB2, PSME3
PKD2	14	6	KIF11, ADH1C, MTAP, ADH1A, PPP3CA, UCHL3
PKD2L1	3	6	MYOF, HNRNPA2B1, LGI1, CAB39, GBF1, PRPS2
PKHD1	1	7	ORM1, CSTF2T, ILK, ATXN2, EML4, BAG2, PTGIS
PPIL6	0	2	AK9, CEP57L1
PPP1CB	26	9	NRBP1, DHX57, GCKR, NCOA1, MATN1, SRD5A2, UPK2, SH3KBP1, ZNF512
PTCH1	65	5	CORO2A, ECM2, DPP3, TMOD1, UFM1
QRFPR	0	9	BBS12, BBS7, NDNF, EXOSC9, ADAD1, KIAA1109, TRPC3, TNIP3, TMEM155
RABL5	0	0	None
ROPN1L	3	4	FAM173B, 42435, CMBL, CCT5
RPGR	11	5	NUDT12, ATP6AP2, USP9X, SLC9A7, TSPAN7
RPGRIP1	23	1	SLC6A2
RPGRIP1L	3	2	CES1P1,SLC6A2
RSPH1	1	7	LINC01547, OSMR, COL18A1, LINC00313, LINC00334, LRRC3, UMODL1
RSPH10B	0	7	DAGLB, CCZ1B, FOXK1, MMD2, RBAKDN, GRID2IP, SLC29A4
RSPH3	5	6	PNLDC1, MRPL18, RGS17, TAGAP, SERAC1, SYTL3
RSPH4A	0	6	LACE1, HS3ST5, FAM26F, FLJ34503, FAM26E, ZUFSP
RSPH6A	0	1	SLC1A5
RSPH9	2	9	C6orf223, LRRC73, OARD1, KLHDC3, ADCY10P1, TSPO2, TCTE1, TAF8, SPATS1
RTDR1	13	0	None
SMO	8	1	LINC-PINT
SPAG1	3	7	MME, NIPAL2, CPQ, POLR2K, RGS22, COL4A1, STK3
SPAG16	2	1	IKZF2
SPAG17	0	2	GDAP2, FAM46C
SPAG6	2	2	OTUD1, PIP4K2A
SPEF2	0	10	C5orf42, BRIX1, NIPBL, LMBRD2, DNAJC21, CAPSL, NADK2, UGT3A1, TARS, WDR70
TCTE1	1	7	KLC4, C6orf223, LRRC73, KLHDC3, SPATS1, TAF8, RSPH9
TCTE3	1	4	TCP10, LINC01558, RPS6KA2, RNASET2
TCTN1	0	3	GLTP, SVOP, FAM222A
TCTN2	0	3	COQ5, KDM2B, CCDC92
TCTN3	0	0	None
TMEM17	0	3	FAM161A, COMMD1, C2orf74
TMEM216	1	9	TEX40, SAC3D1, ATL3, EFEMP2, CABP2, CCDC87, SSH3, POLA2, TRMT112
TMEM231	3	4	TMEM170A, CNTNAP4, ADAT1, VAT1L
TMEM237	2	2	ATP6V1C2, SFXN5
TMEM30B	0	4	BCAR1, SLC38A6, C14orf37, NAA30
TMEM67	1	4	NDUFAF6, INTS8, FAM92A1, LAPTM4B
TRAF3IP1	27	3	ATG4B, ANO7, SNED1
TSG101	86	10	PILRA, RPS13, MYLPF, DNAJB11, CALML5, ST5, PPP2R5D, MAPK15, ZNF160, ZNF419
TTC21B	0	4	COBLL1, G6PC2, CSRNP3, CERS6
TTC28	3	4	GATSL3, TPST2, PES1, PITPNB
TTC30B	0	7	FKBP7, CCDC141, PLEKHA3, RBM45, HNRNPA3, SESTD1, TTC30A
TTC8	1	1	PTPN21
VPS28	27	7	THEM6, CCNB1, OXCT1, OPLAH, RPL37A, SCRIB, CACNA1I
VPS37A	7	4	MICU3, CCAR2, COL11A2, KIAA1456
VPS4B	7	2	SPHAR, PHLPP1
WDR19	0	3	NWD2, TLR10, CHRNA9
WDR35	0	0	None
WDR63	0	2	RPL17P5, RPF1
WDR78	0	1	GNG12
ZMYND10	10	2	ACTR3, RALGDS
ZMYND12	0	3	ERMAP, C1orf50, CABIN1

The table shows the number of known and computationally predicted novel PPIs for each of the 165 cilia genes, and lists their corresponding novel interactors.

For each of the ciliary proteins, we computed the enrichment of gene ontology (GO) terms among its interacting partners in order to aid in the discovery of its function using BinGO (Biological Networks Gene Ontology tool)^43^. This information is especially useful for those ciliary proteins that have either no known or very few known GO biological process terms. For example, there are 11 genes that have no known GO terms, and we predicted new GO terms for each of those genes, for e.g. 27 novel GO terms for ARMC4, 11 for CCDC63, and 30 for DNAAF2.

**Figure 2:**
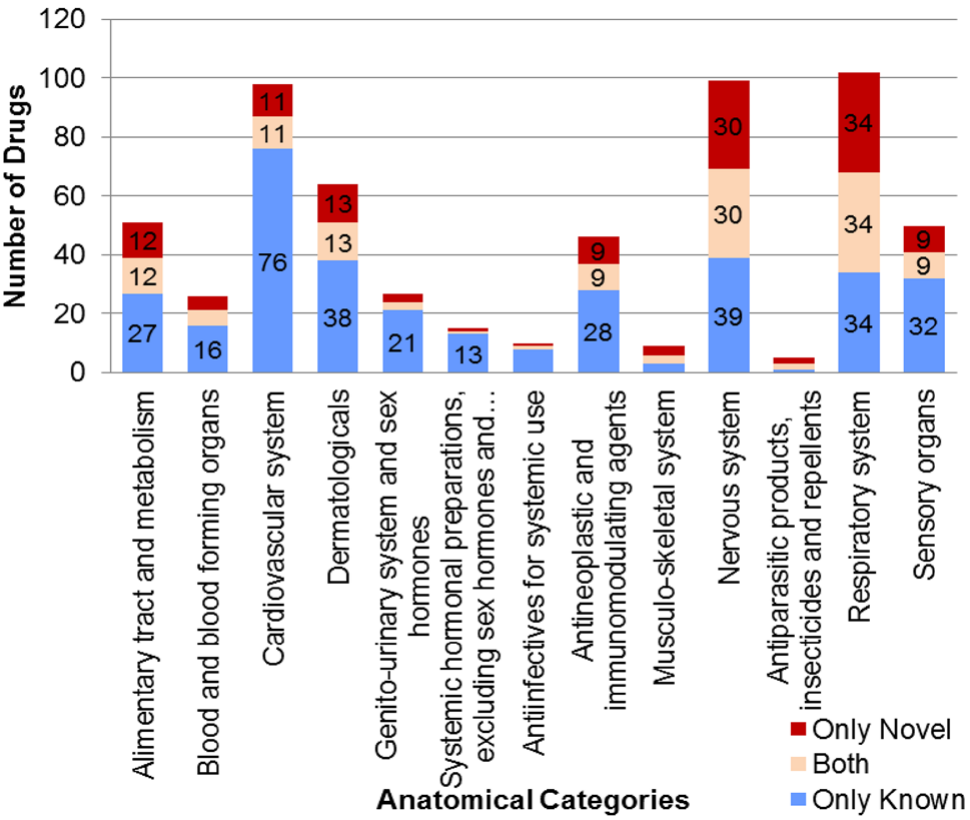
Number of drugs targeting genes in the cilia interactome. The numbers are shown separated by the anatomic category of the drugs (anatomic, therapeutic and chemical classification) and also separated by whether they target known interactors (blue) or novel interactors (red) or both (cream-colored).

We computed the pathway associations of genes in the interactome, using the Ingenuity Pathway Analysis (IPA) suite (Ingenuity Systems, www.ingenuity.com). This showed a significant overlap of neuronal pathways with the cilia interactome (see selected pathways in Table 2). The complete list of all pathways, their *p* values and the genes from the interactome that are associated with these pathways, are given in Supplementary File 3. We also extracted information about drugs targeting the genes in the interactome. This analysis showed that there are several genes that are targets to drugs belonging to the Anatomic category of “nervous system”, highlighting the connection between cilia and the nervous system as shown in Fig. 2 and Supplementary File 4.

**Table 2:** Overlap of neuronal pathways in cilia interactome.

Neuronal pathways in cilia interactome	*p* value	Number of proteins	Proteins
Huntington's disease signaling	1.00E−13	15	PLCB2,SHC1,CASP1,GNG12,PIK3R6,POLR2G,PIK3R4,CLTA,PIK3R2,CAPN1,PDPK1,RPH3A,CASP8,POLR2K,PRKD3
Dopamine-DARPP32 feedback in cAMP Signaling	2.00E−08	8	PLCB2,PPP2R3A,PRKG2,CALML5,PLCD1,PPP2R5D,PPP3CA,PRKD3
CREB signaling in neurons	3.02E−08	11	CALML5,PLCB2,SHC1,GNG12,PIK3R6,POLR2G,PIK3R4,PIK3R2,PLCD1,POLR2K,PRKD3
nNOS signaling in neurons	8.13E−06	4	PPP3CA,CAPN1,CALML5,PRKD3
nNOS signaling in neurons	8.13E−06	4	PPP3CA,CAPN1,CALML5,PRKD3
Axonal guidance signaling	8.91E−06	15	PLCB2,GIT1,SHC1,PIK3R6,ACTR3,NTN3,GNG12,MICAL1,PIK3R4,MYLPF,PLCD1,PPP3CA,PRKD3,SEMA7A,PIK3R2
eNOS signaling	1.17E−05	13	BDKRB1,CALML5,AQP3,CNGA1,CASP8,SLC7A1,HSP90AB1,PIK3R6,PIK3R4,PIK3R2,PDPK1,CHRNA9,PRKD3
Synaptic long term potentiation	1.26E−05	5	PPP3CA,PLCB2,PRKD3,CALML5,PLCD1
Wnt/Beta-catenin signaling	1.62E−05	6	CDKN2A,TLE1,PPP2R3A,ILK,SOX17,PPP2R5D
Neuregulin signaling	3.63E−05	8	SHC1,TMEFF2,HSP90AB1,HBEGF,BAD,PIK3R2,PDPK1,PRKD3
Neuropathic pain signaling in dorsal horn neurons	0.000224	7	PLCB2,FOS,PIK3R6,PIK3R4,PIK3R2,PLCD1,PRKD3
Calcium signaling	0.000603	8	TNNT1,SLC8B1,CABIN1,CALML5,TRPC3,PPP3CA,ATP2B3,CHRNA9
Dopamine receptor signaling	0.00251	3	PPP2R5D,SPR,PPP2R3A
Glutamate receptor signaling	0.00398	1	CALML5
Synaptic long term depression	0.00676	7	PLCB2,PPP2R3A,NPR2,PLCD1,PRKG2,PPP2R5D,PRKD3
Wnt/Ca + pathway	0.0102	3	PPP3CA,PLCB2,PLCD1
Dendritic cell maturation	0.0102	12	PLCB2,STAT4,COL11A2,COL18A1,CD80,TRGV9,IL6,PIK3R6,PIK3R4,IL32,PIK3R2,PLCD1
Reelin signaling in neurons	0.0407	3	PIK3R6,PIK3R4,PIK3R2

Neuronal pathways which were present in cilia interactome with number of novel interactors.

### Experimental Validation of novel cilia PPIs in independent studies

Four of the novels PPIs that we predicted for cilia genes were independently recovered by other groups. TMEM237-SFXN5 and DYNLL2-c17orf47 were recovered by yeast two-hybrid experiments in the recent release of the human protein interactome map^57^. We also predicted two PPIs of IFT140 that were discovered as part of the CPLANE interactome using affinity purification-mass spectrometry, but were not deposited in BioGRID or HPRD: IFT140-TELO2 and IFT140-TRAP1^36^. It is also worth noting that 8 novel interactors in the interactome appeared among the proteins isolated from the primary cilia of mouse kidney cells using a method called MudPIT (multidimensional protein identification technology)^33^: ABCE1, CCDC47, CCT5, G3BP1, GBF1, RAB10, RAN and USP14. 94 genes in the cilia interactome, including 44 cilia genes, 36 known and 14 novel interactors, were also recovered as regulators of the ciliary sonic hedgehog pathway in a CRISPR genetic screen (*p* value = 2.28e−19)^58^. The interactome was also significantly enriched with genes differentially expressed in bronchial biopsies of primary ciliary dyskinesia patients (*p* value = 2.64e−02)^59^.

### Functional interactions of cilia genes with predicted novel interactors

We used ReactomeFIViz^60^, a Cytoscape plugin, to extract known functional interactions between cilia genes and their novel interactors. Five novel PPIs had such functional interactions, namely, IFT57-CLTA, DYNLL2-KIF2B, IFT57-HTT, CHMP5-UBAP1 (‘*part of the same complex*’, ‘*bound by the same set of ligands*’) and IFT57 → CASP8 (‘*activation*’).

## Discussion

We developed the interactome of ciliary proteins that included IFT, BBS, TZ, ciliary membrane proteins and proteins in motile cilia. The interactome includes novel computationally predicted PPIs for multiple proteins, including proteins with few or no previously known PPIs.

Both analysis of individual novel PPIs and the cilia interactome as a whole has the potential to highlight connections to specific neurological disorders and lead to biologically insightful and clinically translatable results. We interpreted the functions of individual novel PPIs using literature-based evidence and top pathways obtained from IPA (See Supplementary File 5 for testable hypotheses on novel PPIs involved in neuropsychiatric disorders, primary ciliary dyskinesia, hydrocephalus and in biological processes such as ciliogenesis and trafficking of membrane receptors in cilia). The following is a demonstrative example of a systems-level analysis.

### Cilia, Alzheimer’s disease and aging

Alzheimer’s disease (AD) is a progressive neurodegenerative disease with an estimated prevalence of 10–30% in the population aged 65 years and more, characterized by memory loss (dementia), behavioral changes, impaired cognition and language^61^. Around two-thirds of dementia cases is attributed to AD^61^. Hippocampus, a region in the brain critical to memory and learning, exhibits signs of neurodegeneration in the early stages of AD^62^. It has been speculated that memory and learning deficits in AD may be associated with aging and reduced neurogenesis in the hippocampus^62–64^. It is interesting to note that primary cilia have been shown to mediate sonic hedgehog signaling (Shh) to regulate hippocampal neurogenesis^65,66^. So, we explored interconnections of AD, aging and cilia in the PPI network (the ‘interactome’), while asking the following questions: Are genes associated with AD, aging and cilia closely connected in the interactome? Will such a network also include genes involved in Shh signaling and neurogenesis, and genes expressed in the hippocampus? What specific biological processes may underlie the connections of AD to aging, and will they interact with the Shh pathway?

**Figure 3:**
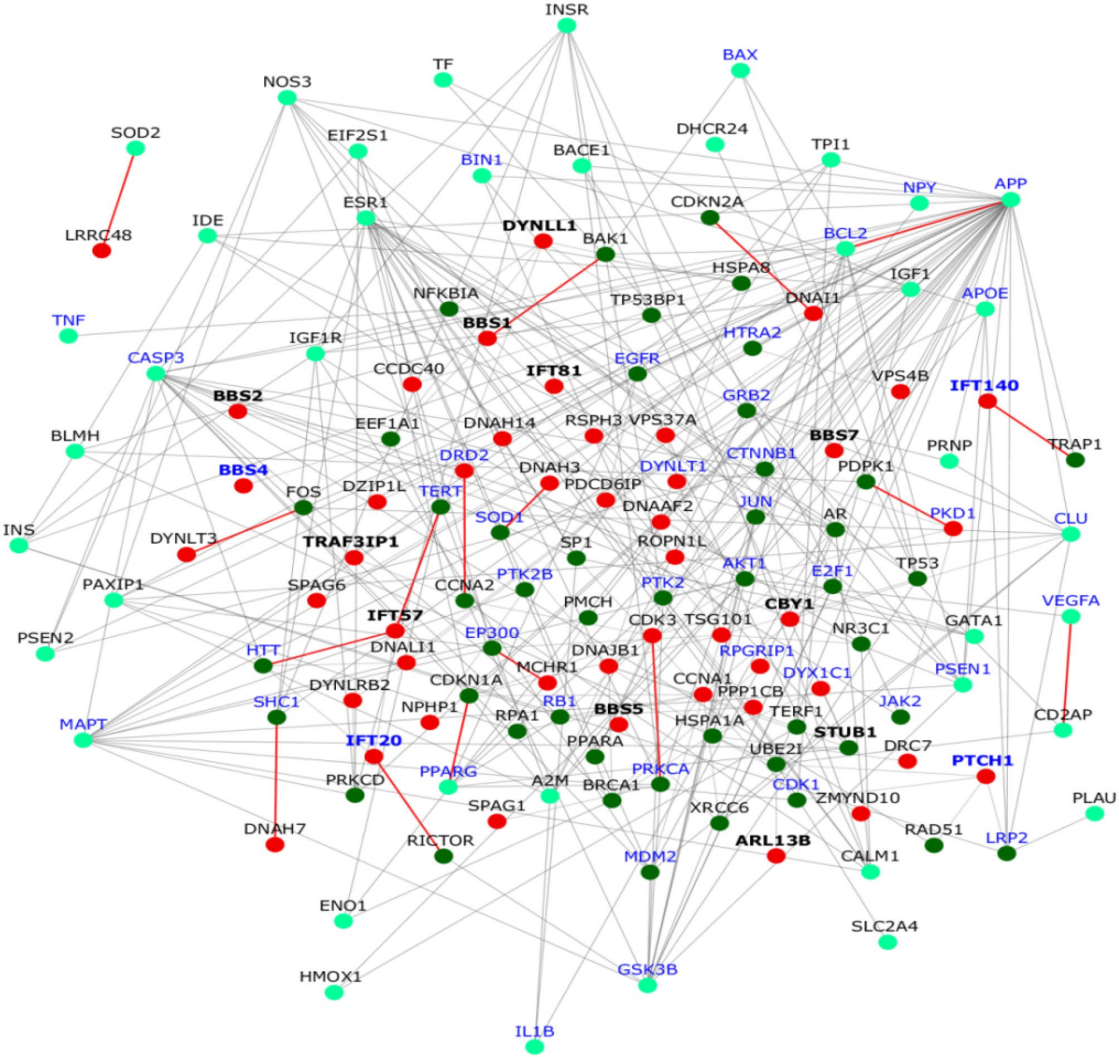
Interconnections between cilia, aging and Alzheimer’s disease genes. Cilia genes are shown as red nodes; AD genes are colored in cyan and aging genes in green. PPIs are shown as edges, where grey edges are known PPIs and red color edges are novel predicted PPIs. Genes with bold labels are involved in the sonic hedgehog (Shh) pathway and those with blue labels are involved in neurogenesis. Note that, in this case, a bold blue-labeled gene indicates a cilia gene with Shh involvement, which is also involved in neurogenesis.

**Figure 4:**
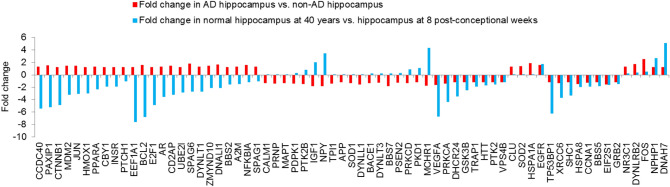
Direction of fold change of gene expression levels in AD vs. non-AD hippocampal samples compared with expression at 40 years vs. 8 post-conceptional weeks. The X-axis shows the 67 genes that are differentially expressed in the hippocampus of Alzheimer’s disease patients compared with the hippocampus of healthy subjects. The red bars on the Y-axis show the fold change of the differential expression of these genes in AD vs. non-AD hippocampal samples. The blue bars on the Y-axis show the fold change in the expression level of these genes in normal hippocampus at 40 years after birth (middle adulthood) compared with 8 weeks after conception (fetal life) in healthy humans.

Significant overlap was found between cilia and the AD interactomes (*p* value = 0.022). The AD interactome was highly significantly enriched in ‘human aging-related genes’ (*p* value = 1.77e−37), compiled from the GenAge database^67^. 51 aging genes co-occurred in AD and cilia interactomes. The subnetwork of these 51 genes and their AD and cilia interactors is shown in Fig. 3. In this subnetwork, aging genes connected cilia genes with/without Shh involvement to AD genes (Fig. 3).The next question we asked was: do any of the 51 genes directly interact with a ciliary gene involved in the Shh pathway? 15 cilia genes in the network were also recovered as regulators of the Shh pathway in a CRISPR genetic screen: ARL13B, BBS1-2, BBS4-5, BBS7, CBY1, DYNLL1, IFT140, IFT20, IFT52, IFT81, PTCH1, STUB1 and TRAF3IP1^58^. These 15 genes had direct interactions with 14 aging genes, 6 AD genes and 2 cilia genes. This included 13 novel predicted interactions connecting aging genes to cilia genes including 4 Shh genes (in *italics*): BAK1-*BBS1*, CDKN2A-DNAI1, TRAP1-*IFT140*, PDPK1-PKD1, SOD1-DNAH3, CCNA2-DRD2, TERT-*IFT57*, HTT-*IFT57*, FOS-DYNLT3, EP300-MCHR1, SHC1-DNAH7, PRKCA-CDK3 and RICTOR-*IFT20*. The network was significantly enriched in the GO term ‘*neurogenesis*’ (*p* value = 5.66e−12) and in genes expressed in the hippocampus (transcripts per million ≥ 2) (*p* value = 2.54e−09). The cilia genes DYNLT1 and PKD1 were associated with neurogenesis, and IFT20, IFT140, PTCH1 and BBS4 were Shh regulators also associated with neurogenesis. Reduced size of hippocampus was noted in mutant mouse models of 5 cilia genes, namely BBS1, BBS2, BBS4, BBS7 and PDCD6IP (Mammalian Phenotype Ontology term: *small hippocampus*)^68–70^. We next identified the biological processes that may be specifically affected in AD in relation to its links with aging. 75 genes in the network were differentially expressed in the hippocampus of AD patients compared with non-AD subjects (GSE48350^71^, GSE36980^72^, GSE1297^73^, GSE28146^49^, GSE29378^48^). We then examined the fold change in the normal expression of these 75 genes in the hippocampus at 40 years compared with 8 post-conceptional weeks. For this, we used the 'developmental transcriptome' from the BrainSpan Atlas containing RNA-Seq data of up to 16 brain regions from post-conceptional weeks (number of weeks elapsed from the first day of the last menstrual period and the day of the delivery) to middle adulthood (up to 40 years)^52^. The genes were grouped based on the specific direction in which their expression varied in AD versus aging (i.e. fold change in same/opposite directions in *AD versus non-AD hippocampal samples* compared with *expression at 40 years versus 8 post-conceptional weeks*) (Fig. 4). 42 genes showed an expression change in the opposite direction in AD versus aging. Out of this, 18 genes were underexpressed in AD but overexpressed in aging; they were enriched in the GO term *‘calcium-mediated signaling*’ (*p* value = 8.72e−09). It has been postulated that calcium signaling pathways involved in cognition may be remodeled by an activated amyloidogenic pathway in AD, resulting in elevated levels of calcium and a constant erasure of new memories through enhancement of mechanisms involved in long term depression^74^. It is also worth noting that Shh signaling requires calcium mobilization^75^. The 18 genes included the cilia genes DYNLL1, DYNLT3, PKD1 and MCHR1, and the ciliary Shh regulator BBS7. 24 genes were overexpressed in AD but underexpressed in aging; they were enriched in ‘*circulatory system development*’ (*p* value = 3.04e−07). Loss of hippocampal blood vessel density accompanied by ultrastructural changes in the blood vessels have been observed in a senescence-accelerated rat model of AD^76^. It is interesting to note that circulatory system processes were found to be upregulated in early stages of AD-like pathology in this model, while they were found to be downregulated with age, similar to our observations^76^. It is also interesting to note that neovascularization requires Shh signaling^77^. The 24 genes included the cilia genes CCDC40, SPAG6, ZMYND10, DNALI1 and SPAG1, BBS2 and CBY1 which are ciliary Shh regulators, DYNLT1, a cilia gene involved in neurogenesis and PTCH1 which is an Shh ligand also involved in neurogenesis. 25 genes showed an expression change in the same direction (either under/overexpression) in AD versus aging including the cilia genes VPS4B, CCNA1, DYNLRB2, NPHP1, DNAH7 and the ciliary Shh regulator BBS5; ‘*negative regulation of cell death*’ was enriched in this group (*p* value = 1.59e−09). Shh maintains neural stem cells in the hippocampus by inhibiting cell death^78^.

In summary, our analysis demonstrates that aging and AD genes directly interact with ciliary Shh regulators in the interactome. This network is enriched in genes associated with neurogenesis and expressed in the hippocampus. Genes involved in *calcium-mediated signaling* and *circulatory system development* are differentially expressed in the opposite direction in AD versus aging, whereas genes involved in *regulation of cell death* are differentially expressed in the same direction.

### Cilia and neuronal pathways and functions

Our pathways enrichment analysis of the cilia interactome revealed several neuronal pathways with high statistical significance (see Table 2 and Supplementary File 3). This included *axonal guidance signaling pathway* with 15 novel cilia interactors, *Huntington disease signaling* with 15 novel interactors, *eNOS signaling pathway* with 13, *Wnt signaling* with 6, *DARPP32 feedback in cAMP signaling* with 8, *dopamine receptor signaling* with 3, *synaptic long-term depression* with 7, and *synaptic long-term potentiation* with 5 novel interactors. Dopamine receptors are localized in the membrane of neuronal cilia^19^, suggesting that these novel cilia interacting partners may have a role in neurotransmission. *Dopamine signaling*, *eNOS signaling*, and *synaptic long term potentiation* pathways are also known to be associated with neuropsychiatric disorders such as schizophrenia^79,80^. The identification of Huntington’s disease (HD) pathway in the cilia interactome is also notable given that the protein huntingtin (HTT) localizes to the centrosome and plays an important role in ciliogenesis. The HD mutant mouse model exhibits abnormal cilia motility and cerebrospinal fluid flow^23^. Recovery of Wnt signaling thought to be involved in schizophrenia etiology is also of interest^81,82^.

Analysis of the known and novel PPIs and GO term associations identified a role for cilia in neuronal disease pathogenesis. While consistent with the known role of cilia in several key processes in the nervous system such as the neuronal signaling and development, these findings reveal novel connections between cilia and these functional modules. The defects in neuronal migration and differentiation are the underlying cause of abnormal neural circuitry in psychiatric disorders^12^. This is further supported by the reported linkage of neuropsychiatric risk genes to cilia^15,19^ and the finding of neuropsychiatric phenotypes and brain abnormalities in ciliopathies^5,12^. Our interactome analysis shows that TCTN2, cilia gene with known role in neuronal development and migration^12^ has 3 novel interactors and neuronal GO terms such as *initiation of neural tube closure*, *midbrain morphogenesis* and *mid brain development* are enriched among the interacting partners. The GO terms that are enriched for interacting partners of ARMC4 include *sympathetic neuron projection guidance*, *axonogenesis*, *axon extension*, and *axon fasciculation*. Dynein gene, DNAAF2 has only one known but 4 predicted interactions. Two of those novel interactors, ATL1 and TRIM9 are shown to be associated with cognitive performance and psychosis respectively through GWAS. The GO terms such as *axonogenesis*, *neuron maturation*, *synaptic growth at neuromuscular junction* are enriched among the interacting partners. Ciliary membrane genes DRD1 and DRD2 that are implicated in neurotransmission and linked to mental illnesses such as schizophrenia^83^ were identified with 4 and 12 novel interactors, respectively; the associated GO terms were *neuronal action potential* and *synaptic plasticity regulation*. We also observed 4 novel interactors for the cilia protein TMEM67, including two proteins associated with cilia assembly, LAPTM4B and NDUFAF6, with NDUFAF6 also known to be associated with Alzheimer’s disease^84^. Both ATG7, a novel interactor of the ciliary protein PDCD6IP, and SPR, a novel interactor of GPR83 have been associated with Parkinson’s disease^85,86^. GIT1, a novel interactor of B9D1 is associated with attention deficit hyperactivity disorder and MME, a novel interactor of SPAG1with Alzheimer’s disease^87^. On inspecting mammalian phenotype ontology (MPO) terms (www.informatics.jax.org/), 42 novel interactors were found to be associated with various morphological or physiological aspects of brain in mice. For example, the novel interactor ITSN1 was associated with *decreased brain size*, *abnormal corpus callosum*, *hippocampal fimbria*, *hippocampal fornix*, *brain white matter* and *anterior commissure morphology*. These findings support the role of these novel interactions and the GO terms in understanding the crucial role played by cilia biology in neuropsychiatric disorders.

### Overlap of cilia and neuropsychiatric disorder interactomes

To examine the connection between cilia and neuropsychiatric disorders, we computed the overlap between their interactomes. We considered 7 neuropsychiatric disorders (NPDs), namely Attention Deficit Hyperactivity Disorder (ADHD), Major Depressive Disorder (MDD), schizophrenia, bipolar disorder, autism spectrum disorder, Alzheimer’s disease and Parkinson’s disease. We extracted the genes associated with each disorder from the GWAS catalog (www.ebi.ac.uk/gwas/) and then assembled disorder-specific interactomes with known PPIs from HPRD and BioGRID. We then computed how closely connected the cilia genes are to NPD genes by computing how many genes or interactors were shared between the cilia interactome and each NPD interactome. This analysis showed the overlap to be statistically significant (Table 3). For example, cilia interactome has an overlap of 88 genes with ADHD interactome (*p* value = 1.2E−16) of which 17 are novel interactors of cilia. Similar comparisons with other NPDs also showed overlaps as shown in Table 3.

**Table 3:** Overlap of neuropsychiatric disease (NPD) interactomes and genes differentially expressed in NPDs with the cilia interactome.

NPD	NPD interactome size	*p* value of overlap	# Genes common to both interactomes	# Novel interactors common to both	# Genes differentially expressed in the disorder	# Genes differentially expressed in the cilia interactome	*p* value of overlap	# Differentially expressed novel genes
ADHD	406	1.20E−16	88	17	n/a	n/a	n/a	n/a
Alzheimer's disease	417	2.20E−24	104	19	1,103	106	4.70E−05	46
Autism spectrum disorder	53	2.20E−05	15	5	3,692	314	2.40E−04	119
Bipolar disorder	764	3.10E−29	163	31	1,188	101	3.40E−02	38
Major depressive disorder	974	3.70E−23	177	32	187	21	2.50E−02	8
Parkinson's disease	520	1.20E−20	112	18	2,487	258	6.20E−03	104
Schizophrenia	688	1.50E−16	125	26	1,320	118	5.60E−03	40
Intellectual disability	n/a	n/a	n/a	n/a	706	75	6.50E−03	32

The significance of the overlap along with the number of genes common to the NPD interactome/expression datasets and the cilia interactome are shown.

### Overlap of cilia interactome with genes differentially expressed in neuropsychiatric disorders

965 genes in the cilia interactome were found to be expressed (transcripts per million ≥ 2) in several brain regions including amygdala, anterior cingulate cortex, caudate, cerebellum, frontal cortex, hippocampus, hypothalamus, nucleus accumbens, putamen, spinal cord and substantia nigra, from GTEx RNA-Seq data^51^ (*p* value = 3.93E−58). Novel interactors of cilia genes were found to be highly statistically enriched among these genes expressed in the human brain (*p* value = 8.14E−09).

We then computed the overlap of genes differentially expressed in neuropsychiatric disorders with the genes in the cilia interactome. We analyzed gene expression datasets of MDD (GSE53987)^44^, schizophrenia (GSE17612)^45^, bipolar disorder (GSE12679)^46^, autism spectrum disorder (GSE18123)^47^, Alzheimer’s disease (GSE29378)^48^, Parkinson’s disease (GSE28894) and non-syndromic intellectual disability (GSE39326)^50^. The analysis showed the overlap to be statistically significant (Table 3). For example, the cilia interactome has an overlap of 106 genes with genes differentially in the Alzheimer’s disease dataset (*p* value = 4.7E−05) of which 46 are novel interactors of cilia.

### Cilia and nervous system drug targets

Given the strong connection between the cilia interactome and neuronal pathways, we tested the possibility of repurposing drugs targeting proteins in the cilia interactome for treating neurological disorders. Identifying new uses for drugs shortens the time of drug discovery and approval^88^. For example, the drug amantadine which is used to treat influenza infection was successfully repurposed to treat dyskinesia and Parkinson’s disease^88^. This analysis identified 548 drugs targeting 184 genes in the cilia interactome. These fall into 3 major Anatomic Therapeutic Chemical (ATC) classification system categories, nervous system with 99 drugs, 102 drugs in the respiratory system, and 98 drugs in the cardiovascular system (Fig. 2, Supplementary File 4). This finding points at therapeutics targeting the cilia proteins which may provide a novel strategy for treating neurological disorders.

Overall, 76 nervous system drugs targeted 7 novel interactors: HRH1, SLC6A2, CHRNA9, NQO2, ORM1, CACNA1I and CACNA1G. 57 drugs targeting 22 genes in the interactome are used in the treatment of at least one among the following neurological disorders- Parkinson’s disease, Alzheimer’s disease, attention deficit hyperactivity disorder (ADHD), major depressive disorder (MDD), autism spectrum disorder, schizophrenia and bipolar disorder- out of which 35 drugs target 6 novel interactors, namely CACNA1G, CACNA1I, CHRNA9, HRH1, SLC6A2 and ORM1. 10 out of these 57 drugs targeted cilia genes as well as known and novel interactors of cilia genes: asenapine, chlorpromazine, clozapine, loxapine and paliperidone are schizophrenia drugs, olanzapine is used in the treatment of Alzheimer’s disease and schizophrenia, amphetamine in ADHD, imipramine in ADHD and MDD, mirtazapine in MDD and nortriptyline in schizophrenia, ADHD, MDD and bipolar disorder.

Among other novel interactors targeted by nervous system drugs is SLC6A2 which is involved in neurotransmission and is associated with ADHD^89,90^. SLC6A2 interacts with RPGRIP1L, a ciliary protein known to cause Joubert syndrome, MKS and bipolar disorder^91,92^. The novel interactors CACNA1I and CACNA1G targeted by nervous system drugs are calcium channels that are known to be associated with Alzheimer’s disease and schizophrenia, respectively^93,94^. These novel interactors which are drug targets may have significant impact on the nervous system, and the pathogenesis of neurological disorders.

In an independent study, we proposed that the drug acetazolamide which targets the genes CA2 and CA4, having known interactions with the cilia genes, DYNLL1 and CDK3 respectively, may be repurposed for schizophrenia based on negative correlation of drug-induced versus disease-associated gene expression profiles and other biological evidences^95^. Acetazolamide is currently under consideration for funding for clinical trial. Several cancer drugs with reported effects on ciliogenesis target known and novel interactors in the cilia interactome. Vinblastine targeting JUNN, a known interactor of BBS7 and TSG101, and TUBB, a known interactor of NPHP1 and DYNLL1, inhibits cilia regeneration in partially deciliated *Tetrahymena* (a unicellular ciliate)^96^. Valproic acid targeting HDAC9, a known interactor of PKD1, restores ciliogenesis in pancreatic ductal adenocarcinoma cells^97^. Gefitinib targeting EGFR, a known interactor of PDCD6IP, inhibits the smoking-induced loss of ciliated cells in the airway^98^. Gefitinib also increases the percentage of ciliated cells in human pancreatic cancer cell lines^99^. Geldanamycin targeting HSP90AB1, a novel interactor of CETN3, induces lengthening of cilia in 3T3-L1, a fibroblast cell line^100^.

## Conclusion

We identified novel PPIs of cilia proteins and their associated pathways, their enriched Gene Ontology term associations, and drugs that target the interactors. This cilia interactome analysis reveals a link between cilia function, neuronal function and neurological disorders. We also demonstrated the interconnections of Alzheimer’s disease, cilia and aging genes. The predicted interactions will have to be validated at the level of network perturbations in the disease state by comparing neuropsychiatric patients with healthy controls. However, one has to be aware of a few caveats while studying the role of ciliary genes in neuropsychiatric disorders (NPDs). Association of a ciliary gene with a NPD can be unequivocally ascertained only if this association is discovered within the ciliary compartment in the context of the particular NPD, i.e. a mechanistic link between ciliary function and the disorder has to be demonstrated. It may not be a true association if a ciliary gene was shown to be associated with a NPD in a cellular context not connected with cilia; a protein may perform its function at different subcellular locations. Mapping the interactome of cilia genes would be useful in carrying out network-based systems biology studies, which will help elucidate the contribution of these novel PPIs to nervous system disease pathology as well as to develop novel therapeutics for these disorders.

## Supplementary information

Supplementary Information 1.

Supplementary Information2.

Supplementary Information 3.

Supplementary Information 4.

Supplementary Information 5.

Supplementary Information 6.

Supplementary Information 7.

## Data Availability

We will make the cilia interactome publicly available on our web application Wiki-Pi^101^. Novel PPIs will be highlighted in yellow on the website. The number of novel and known PPIs of the cilia genes are given in Supplementary File 1. Interactome network diagram that is shown in Fig. 1 is also being made available in PDF format and in Cytoscape file format as Supplementary File 6 and Supplementary File 7 respectively. PDF file would be suitable for printing in high resolution and for electronically searching for specific genes, and Cytoscape would allow further processing and data analysis. Wiki-Pi allows users to search for interactions by specifying biomedical associations of one or both proteins involved. Thus, queries can be customized to include/exclude gene symbol, gene name, GO annotations, diseases, drugs, and/or pathways for either gene involved in an interaction. For example, researchers can search for interactions by giving at least one cilia gene and a pathway of interest, say “IFT20 interactions where the interactor is involved in *immunity*”; this query would match 5 PPIs out of a total of 19 PPIs of IFT20. Another example is the search “find interactions where one protein’s annotation contains the word *ciliary* and the other protein’s annotation contain the word *neuronal*”. The search returns 353 PPIs, out of which 13 are novel PPIs.

## Abbreviations

PPI: Protein–protein interaction
GO: Gene ontology
HiPPIP: High-confidence protein–protein interaction prediction model
