# Supplementary material for: Cilia interactome with predicted protein–protein interactions reveals connections to Alzheimer’s disease, aging and other neuropsychiatric processes
Source: Sci Rep. 2020 Sep 24;10:15629. doi: 10.1038/s41598-020-72024-4 (PMC7515907; doi:10.1038/s41598-020-72024-4)
Supplement: Supplementary file 1 — Supplementary Information 1. [file 41598_2020_72024_MOESM1_ESM.docx]

**Cilia interactome with predicted protein-protein interactions reveals connections to Alzheimer’s disease, Aging and other Neuropsychiatric Processes**

# Kalyani B. Karunakaran^1^^[[1]](#footnote-1)^, Srilakshmi Chaparala^2,3†^, Cecilia W. Lo^4^ and Madhavi K. Ganapathiraju^2,5^*

# ^1^Indian Institute of Science, Bangalore, India

^2^Department of Biomedical Informatics, ^3^Health Sciences Library System, and ^4^Department of Developmental Biology, School of Medicine, and ^5^Intelligent Systems Program, School of Computing and Information, University of Pittsburgh, Pittsburgh, PA, USA

# *To whom correspondence should be addressed: Madhavi K. Ganapathiraju, Department of Biomedical Informatics, University of Pittsburgh, Pittsburgh, PA 15206, USA. [madhavi@pitt.edu](mailto:madhavi@pitt.edu)

Supplementary File 1: Gene list

Table shows the list of cilia associated genes that were included in this study. HUGO standard gene symbol and Entrez id are shown in the first two columns. Next 2 columns show the number of known and novel (predicted) PPIs.

| **Hugo Symbol** | **Entrez ID** | **Known PPIs** | **Novel PPIs** |
| --- | --- | --- | --- |
| [AHI1](http://severus.dbmi.pitt.edu/wiki-pi-cilia/gene/view/54806) | [54806](http://severus.dbmi.pitt.edu/wiki-pi-cilia/gene/view/54806) | [1](http://severus.dbmi.pitt.edu/wiki-pi-cilia/search?q=AHI1) | [2](http://severus.dbmi.pitt.edu/wiki-pi-cilia/search?q=AHI1) |
| [AK7](http://severus.dbmi.pitt.edu/wiki-pi-cilia/gene/view/122481) | [122481](http://severus.dbmi.pitt.edu/wiki-pi-cilia/gene/view/122481) | [0](http://severus.dbmi.pitt.edu/wiki-pi-cilia/search?q=AK7) | [3](http://severus.dbmi.pitt.edu/wiki-pi-cilia/search?q=AK7) |
| [ARL13B](http://severus.dbmi.pitt.edu/wiki-pi-cilia/gene/view/200894) | [200894](http://severus.dbmi.pitt.edu/wiki-pi-cilia/gene/view/200894) | [1](http://severus.dbmi.pitt.edu/wiki-pi-cilia/search?q=ARL13B) | [4](http://severus.dbmi.pitt.edu/wiki-pi-cilia/search?q=ARL13B) |
| [ARMC4](http://severus.dbmi.pitt.edu/wiki-pi-cilia/gene/view/55130) | [55130](http://severus.dbmi.pitt.edu/wiki-pi-cilia/gene/view/55130) | [3](http://severus.dbmi.pitt.edu/wiki-pi-cilia/search?q=ARMC4) | [3](http://severus.dbmi.pitt.edu/wiki-pi-cilia/search?q=ARMC4) |
| [ATP8A2](http://severus.dbmi.pitt.edu/wiki-pi-cilia/gene/view/51761) | [51761](http://severus.dbmi.pitt.edu/wiki-pi-cilia/gene/view/51761) | [1](http://severus.dbmi.pitt.edu/wiki-pi-cilia/search?q=ATP8A2) | [3](http://severus.dbmi.pitt.edu/wiki-pi-cilia/search?q=ATP8A2) |
| [ATXN10](http://severus.dbmi.pitt.edu/wiki-pi-cilia/gene/view/25814) | [25814](http://severus.dbmi.pitt.edu/wiki-pi-cilia/gene/view/25814) | [9](http://severus.dbmi.pitt.edu/wiki-pi-cilia/search?q=ATXN10) | [9](http://severus.dbmi.pitt.edu/wiki-pi-cilia/search?q=ATXN10) |
| [B9D1](http://severus.dbmi.pitt.edu/wiki-pi-cilia/gene/view/27077) | [27077](http://severus.dbmi.pitt.edu/wiki-pi-cilia/gene/view/27077) | [7](http://severus.dbmi.pitt.edu/wiki-pi-cilia/search?q=B9D1) | [3](http://severus.dbmi.pitt.edu/wiki-pi-cilia/search?q=B9D1) |
| [B9D2](http://severus.dbmi.pitt.edu/wiki-pi-cilia/gene/view/80776) | [80776](http://severus.dbmi.pitt.edu/wiki-pi-cilia/gene/view/80776) | [2](http://severus.dbmi.pitt.edu/wiki-pi-cilia/search?q=B9D2) | [7](http://severus.dbmi.pitt.edu/wiki-pi-cilia/search?q=B9D2) |
| [BBIP1](http://severus.dbmi.pitt.edu/wiki-pi-cilia/gene/view/92482) | [92482](http://severus.dbmi.pitt.edu/wiki-pi-cilia/gene/view/92482) | [0](http://severus.dbmi.pitt.edu/wiki-pi-cilia/search?q=BBIP1) | [0](http://severus.dbmi.pitt.edu/wiki-pi-cilia/search?q=BBIP1) |
| [BBS1](http://severus.dbmi.pitt.edu/wiki-pi-cilia/gene/view/582) | [582](http://severus.dbmi.pitt.edu/wiki-pi-cilia/gene/view/582) | [13](http://severus.dbmi.pitt.edu/wiki-pi-cilia/search?q=BBS1) | [6](http://severus.dbmi.pitt.edu/wiki-pi-cilia/search?q=BBS1) |
| [BBS2](http://severus.dbmi.pitt.edu/wiki-pi-cilia/gene/view/583) | [583](http://severus.dbmi.pitt.edu/wiki-pi-cilia/gene/view/583) | [10](http://severus.dbmi.pitt.edu/wiki-pi-cilia/search?q=BBS2) | [11](http://severus.dbmi.pitt.edu/wiki-pi-cilia/search?q=BBS2) |
| [BBS4](http://severus.dbmi.pitt.edu/wiki-pi-cilia/gene/view/585) | [585](http://severus.dbmi.pitt.edu/wiki-pi-cilia/gene/view/585) | [13](http://severus.dbmi.pitt.edu/wiki-pi-cilia/search?q=BBS4) | [6](http://severus.dbmi.pitt.edu/wiki-pi-cilia/search?q=BBS4) |
| [BBS5](http://severus.dbmi.pitt.edu/wiki-pi-cilia/gene/view/129880) | [129880](http://severus.dbmi.pitt.edu/wiki-pi-cilia/gene/view/129880) | [5](http://severus.dbmi.pitt.edu/wiki-pi-cilia/search?q=BBS5) | [11](http://severus.dbmi.pitt.edu/wiki-pi-cilia/search?q=BBS5) |
| [BBS7](http://severus.dbmi.pitt.edu/wiki-pi-cilia/gene/view/55212) | [55212](http://severus.dbmi.pitt.edu/wiki-pi-cilia/gene/view/55212) | [5](http://severus.dbmi.pitt.edu/wiki-pi-cilia/search?q=BBS7) | [8](http://severus.dbmi.pitt.edu/wiki-pi-cilia/search?q=BBS7) |
| [BBS9](http://severus.dbmi.pitt.edu/wiki-pi-cilia/gene/view/27241) | [27241](http://severus.dbmi.pitt.edu/wiki-pi-cilia/gene/view/27241) | [0](http://severus.dbmi.pitt.edu/wiki-pi-cilia/search?q=BBS9) | [6](http://severus.dbmi.pitt.edu/wiki-pi-cilia/search?q=BBS9) |
| [CASC1](http://severus.dbmi.pitt.edu/wiki-pi-cilia/gene/view/55259) | [55259](http://severus.dbmi.pitt.edu/wiki-pi-cilia/gene/view/55259) | [1](http://severus.dbmi.pitt.edu/wiki-pi-cilia/search?q=CASC1) | [2](http://severus.dbmi.pitt.edu/wiki-pi-cilia/search?q=CASC1) |
| [CBY1](http://severus.dbmi.pitt.edu/wiki-pi-cilia/gene/view/25776) | [25776](http://severus.dbmi.pitt.edu/wiki-pi-cilia/gene/view/25776) | [8](http://severus.dbmi.pitt.edu/wiki-pi-cilia/search?q=CBY1) | [3](http://severus.dbmi.pitt.edu/wiki-pi-cilia/search?q=CBY1) |
| [CC2D2A](http://severus.dbmi.pitt.edu/wiki-pi-cilia/gene/view/57545) | [57545](http://severus.dbmi.pitt.edu/wiki-pi-cilia/gene/view/57545) | [0](http://severus.dbmi.pitt.edu/wiki-pi-cilia/search?q=CC2D2A) | [3](http://severus.dbmi.pitt.edu/wiki-pi-cilia/search?q=CC2D2A) |
| [CCDC114](http://severus.dbmi.pitt.edu/wiki-pi-cilia/gene/view/93233) | [93233](http://severus.dbmi.pitt.edu/wiki-pi-cilia/gene/view/93233) | [8](http://severus.dbmi.pitt.edu/wiki-pi-cilia/search?q=CCDC114) | [4](http://severus.dbmi.pitt.edu/wiki-pi-cilia/search?q=CCDC114) |
| [CCDC135](http://severus.dbmi.pitt.edu/wiki-pi-cilia/gene/view/84229) | [84229](http://severus.dbmi.pitt.edu/wiki-pi-cilia/gene/view/84229) | [6](http://severus.dbmi.pitt.edu/wiki-pi-cilia/search?q=CCDC135) | [0](http://severus.dbmi.pitt.edu/wiki-pi-cilia/search?q=CCDC135) |
| [CCDC39](http://severus.dbmi.pitt.edu/wiki-pi-cilia/gene/view/339829) | [339829](http://severus.dbmi.pitt.edu/wiki-pi-cilia/gene/view/339829) | [0](http://severus.dbmi.pitt.edu/wiki-pi-cilia/search?q=CCDC39) | [1](http://severus.dbmi.pitt.edu/wiki-pi-cilia/search?q=CCDC39) |
| [CCDC40](http://severus.dbmi.pitt.edu/wiki-pi-cilia/gene/view/55036) | [55036](http://severus.dbmi.pitt.edu/wiki-pi-cilia/gene/view/55036) | [1](http://severus.dbmi.pitt.edu/wiki-pi-cilia/search?q=CCDC40) | [3](http://severus.dbmi.pitt.edu/wiki-pi-cilia/search?q=CCDC40) |
| [CCDC63](http://severus.dbmi.pitt.edu/wiki-pi-cilia/gene/view/160762) | [160762](http://severus.dbmi.pitt.edu/wiki-pi-cilia/gene/view/160762) | [0](http://severus.dbmi.pitt.edu/wiki-pi-cilia/search?q=CCDC63) | [2](http://severus.dbmi.pitt.edu/wiki-pi-cilia/search?q=CCDC63) |
| [CCDC65](http://severus.dbmi.pitt.edu/wiki-pi-cilia/gene/view/85478) | [85478](http://severus.dbmi.pitt.edu/wiki-pi-cilia/gene/view/85478) | [0](http://severus.dbmi.pitt.edu/wiki-pi-cilia/search?q=CCDC65) | [3](http://severus.dbmi.pitt.edu/wiki-pi-cilia/search?q=CCDC65) |
| [CCNA1](http://severus.dbmi.pitt.edu/wiki-pi-cilia/gene/view/8900) | [8900](http://severus.dbmi.pitt.edu/wiki-pi-cilia/gene/view/8900) | [32](http://severus.dbmi.pitt.edu/wiki-pi-cilia/search?q=CCNA1) | [9](http://severus.dbmi.pitt.edu/wiki-pi-cilia/search?q=CCNA1) |
| [CDK3](http://severus.dbmi.pitt.edu/wiki-pi-cilia/gene/view/1018) | [1018](http://severus.dbmi.pitt.edu/wiki-pi-cilia/gene/view/1018) | [17](http://severus.dbmi.pitt.edu/wiki-pi-cilia/search?q=CDK3) | [11](http://severus.dbmi.pitt.edu/wiki-pi-cilia/search?q=CDK3) |
| [CEP290](http://severus.dbmi.pitt.edu/wiki-pi-cilia/gene/view/80184) | [80184](http://severus.dbmi.pitt.edu/wiki-pi-cilia/gene/view/80184) | [2](http://severus.dbmi.pitt.edu/wiki-pi-cilia/search?q=CEP290) | [0](http://severus.dbmi.pitt.edu/wiki-pi-cilia/search?q=CEP290) |
| [CETN1](http://severus.dbmi.pitt.edu/wiki-pi-cilia/gene/view/1068) | [1068](http://severus.dbmi.pitt.edu/wiki-pi-cilia/gene/view/1068) | [4](http://severus.dbmi.pitt.edu/wiki-pi-cilia/search?q=CETN1) | [10](http://severus.dbmi.pitt.edu/wiki-pi-cilia/search?q=CETN1) |
| [CETN2](http://severus.dbmi.pitt.edu/wiki-pi-cilia/gene/view/1069) | [1069](http://severus.dbmi.pitt.edu/wiki-pi-cilia/gene/view/1069) | [9](http://severus.dbmi.pitt.edu/wiki-pi-cilia/search?q=CETN2) | [12](http://severus.dbmi.pitt.edu/wiki-pi-cilia/search?q=CETN2) |
| [CETN3](http://severus.dbmi.pitt.edu/wiki-pi-cilia/gene/view/1070) | [1070](http://severus.dbmi.pitt.edu/wiki-pi-cilia/gene/view/1070) | [6](http://severus.dbmi.pitt.edu/wiki-pi-cilia/search?q=CETN3) | [11](http://severus.dbmi.pitt.edu/wiki-pi-cilia/search?q=CETN3) |
| [CHMP5](http://severus.dbmi.pitt.edu/wiki-pi-cilia/gene/view/51510) | [51510](http://severus.dbmi.pitt.edu/wiki-pi-cilia/gene/view/51510) | [14](http://severus.dbmi.pitt.edu/wiki-pi-cilia/search?q=CHMP5) | [8](http://severus.dbmi.pitt.edu/wiki-pi-cilia/search?q=CHMP5) |
| [DAW1](http://severus.dbmi.pitt.edu/wiki-pi-cilia/gene/view/164781) | [164781](http://severus.dbmi.pitt.edu/wiki-pi-cilia/gene/view/164781) | [2](http://severus.dbmi.pitt.edu/wiki-pi-cilia/search?q=DAW1) | [1](http://severus.dbmi.pitt.edu/wiki-pi-cilia/search?q=DAW1) |
| [DDI2](http://severus.dbmi.pitt.edu/wiki-pi-cilia/gene/view/84301) | [84301](http://severus.dbmi.pitt.edu/wiki-pi-cilia/gene/view/84301) | [0](http://severus.dbmi.pitt.edu/wiki-pi-cilia/search?q=DDI2) | [3](http://severus.dbmi.pitt.edu/wiki-pi-cilia/search?q=DDI2) |
| [DNAAF1](http://severus.dbmi.pitt.edu/wiki-pi-cilia/gene/view/123872) | [123872](http://severus.dbmi.pitt.edu/wiki-pi-cilia/gene/view/123872) | [0](http://severus.dbmi.pitt.edu/wiki-pi-cilia/search?q=DNAAF1) | [2](http://severus.dbmi.pitt.edu/wiki-pi-cilia/search?q=DNAAF1) |
| [DNAAF2](http://severus.dbmi.pitt.edu/wiki-pi-cilia/gene/view/55172) | [55172](http://severus.dbmi.pitt.edu/wiki-pi-cilia/gene/view/55172) | [1](http://severus.dbmi.pitt.edu/wiki-pi-cilia/search?q=DNAAF2) | [4](http://severus.dbmi.pitt.edu/wiki-pi-cilia/search?q=DNAAF2) |
| [DNAAF3](http://severus.dbmi.pitt.edu/wiki-pi-cilia/gene/view/352909) | [352909](http://severus.dbmi.pitt.edu/wiki-pi-cilia/gene/view/352909) | [0](http://severus.dbmi.pitt.edu/wiki-pi-cilia/search?q=DNAAF3) | [7](http://severus.dbmi.pitt.edu/wiki-pi-cilia/search?q=DNAAF3) |
| [DNAH1](http://severus.dbmi.pitt.edu/wiki-pi-cilia/gene/view/25981) | [25981](http://severus.dbmi.pitt.edu/wiki-pi-cilia/gene/view/25981) | [0](http://severus.dbmi.pitt.edu/wiki-pi-cilia/search?q=DNAH1) | [7](http://severus.dbmi.pitt.edu/wiki-pi-cilia/search?q=DNAH1) |
| [DNAH10](http://severus.dbmi.pitt.edu/wiki-pi-cilia/gene/view/196385) | [196385](http://severus.dbmi.pitt.edu/wiki-pi-cilia/gene/view/196385) | [0](http://severus.dbmi.pitt.edu/wiki-pi-cilia/search?q=DNAH10) | [3](http://severus.dbmi.pitt.edu/wiki-pi-cilia/search?q=DNAH10) |
| [DNAH11](http://severus.dbmi.pitt.edu/wiki-pi-cilia/gene/view/8701) | [8701](http://severus.dbmi.pitt.edu/wiki-pi-cilia/gene/view/8701) | [0](http://severus.dbmi.pitt.edu/wiki-pi-cilia/search?q=DNAH11) | [3](http://severus.dbmi.pitt.edu/wiki-pi-cilia/search?q=DNAH11) |
| [DNAH12](http://severus.dbmi.pitt.edu/wiki-pi-cilia/gene/view/201625) | [201625](http://severus.dbmi.pitt.edu/wiki-pi-cilia/gene/view/201625) | [0](http://severus.dbmi.pitt.edu/wiki-pi-cilia/search?q=DNAH12) | [1](http://severus.dbmi.pitt.edu/wiki-pi-cilia/search?q=DNAH12) |
| [DNAH14](http://severus.dbmi.pitt.edu/wiki-pi-cilia/gene/view/127602) | [127602](http://severus.dbmi.pitt.edu/wiki-pi-cilia/gene/view/127602) | [1](http://severus.dbmi.pitt.edu/wiki-pi-cilia/search?q=DNAH14) | [6](http://severus.dbmi.pitt.edu/wiki-pi-cilia/search?q=DNAH14) |
| [DNAH17](http://severus.dbmi.pitt.edu/wiki-pi-cilia/gene/view/8632) | [8632](http://severus.dbmi.pitt.edu/wiki-pi-cilia/gene/view/8632) | [0](http://severus.dbmi.pitt.edu/wiki-pi-cilia/search?q=DNAH17) | [0](http://severus.dbmi.pitt.edu/wiki-pi-cilia/search?q=DNAH17) |
| [DNAH2](http://severus.dbmi.pitt.edu/wiki-pi-cilia/gene/view/146754) | [146754](http://severus.dbmi.pitt.edu/wiki-pi-cilia/gene/view/146754) | [0](http://severus.dbmi.pitt.edu/wiki-pi-cilia/search?q=DNAH2) | [6](http://severus.dbmi.pitt.edu/wiki-pi-cilia/search?q=DNAH2) |
| [DNAH3](http://severus.dbmi.pitt.edu/wiki-pi-cilia/gene/view/55567) | [55567](http://severus.dbmi.pitt.edu/wiki-pi-cilia/gene/view/55567) | [0](http://severus.dbmi.pitt.edu/wiki-pi-cilia/search?q=DNAH3) | [7](http://severus.dbmi.pitt.edu/wiki-pi-cilia/search?q=DNAH3) |
| [DNAH5](http://severus.dbmi.pitt.edu/wiki-pi-cilia/gene/view/1767) | [1767](http://severus.dbmi.pitt.edu/wiki-pi-cilia/gene/view/1767) | [0](http://severus.dbmi.pitt.edu/wiki-pi-cilia/search?q=DNAH5) | [13](http://severus.dbmi.pitt.edu/wiki-pi-cilia/search?q=DNAH5) |
| [DNAH6](http://severus.dbmi.pitt.edu/wiki-pi-cilia/gene/view/1768) | [1768](http://severus.dbmi.pitt.edu/wiki-pi-cilia/gene/view/1768) | [0](http://severus.dbmi.pitt.edu/wiki-pi-cilia/search?q=DNAH6) | [1](http://severus.dbmi.pitt.edu/wiki-pi-cilia/search?q=DNAH6) |
| [DNAH7](http://severus.dbmi.pitt.edu/wiki-pi-cilia/gene/view/56171) | [56171](http://severus.dbmi.pitt.edu/wiki-pi-cilia/gene/view/56171) | [2](http://severus.dbmi.pitt.edu/wiki-pi-cilia/search?q=DNAH7) | [9](http://severus.dbmi.pitt.edu/wiki-pi-cilia/search?q=DNAH7) |
| [DNAH8](http://severus.dbmi.pitt.edu/wiki-pi-cilia/gene/view/1769) | [1769](http://severus.dbmi.pitt.edu/wiki-pi-cilia/gene/view/1769) | [0](http://severus.dbmi.pitt.edu/wiki-pi-cilia/search?q=DNAH8) | [3](http://severus.dbmi.pitt.edu/wiki-pi-cilia/search?q=DNAH8) |
| [DNAH9](http://severus.dbmi.pitt.edu/wiki-pi-cilia/gene/view/1770) | [1770](http://severus.dbmi.pitt.edu/wiki-pi-cilia/gene/view/1770) | [0](http://severus.dbmi.pitt.edu/wiki-pi-cilia/search?q=DNAH9) | [3](http://severus.dbmi.pitt.edu/wiki-pi-cilia/search?q=DNAH9) |
| [DNAI1](http://severus.dbmi.pitt.edu/wiki-pi-cilia/gene/view/27019) | [27019](http://severus.dbmi.pitt.edu/wiki-pi-cilia/gene/view/27019) | [2](http://severus.dbmi.pitt.edu/wiki-pi-cilia/search?q=DNAI1) | [6](http://severus.dbmi.pitt.edu/wiki-pi-cilia/search?q=DNAI1) |
| [DNAI2](http://severus.dbmi.pitt.edu/wiki-pi-cilia/gene/view/64446) | [64446](http://severus.dbmi.pitt.edu/wiki-pi-cilia/gene/view/64446) | [0](http://severus.dbmi.pitt.edu/wiki-pi-cilia/search?q=DNAI2) | [1](http://severus.dbmi.pitt.edu/wiki-pi-cilia/search?q=DNAI2) |
| [DNAJB1](http://severus.dbmi.pitt.edu/wiki-pi-cilia/gene/view/3337) | [3337](http://severus.dbmi.pitt.edu/wiki-pi-cilia/gene/view/3337) | [28](http://severus.dbmi.pitt.edu/wiki-pi-cilia/search?q=DNAJB1) | [10](http://severus.dbmi.pitt.edu/wiki-pi-cilia/search?q=DNAJB1) |
| [DNAJB13](http://severus.dbmi.pitt.edu/wiki-pi-cilia/gene/view/374407) | [374407](http://severus.dbmi.pitt.edu/wiki-pi-cilia/gene/view/374407) | [0](http://severus.dbmi.pitt.edu/wiki-pi-cilia/search?q=DNAJB13) | [4](http://severus.dbmi.pitt.edu/wiki-pi-cilia/search?q=DNAJB13) |
| [DNAL1](http://severus.dbmi.pitt.edu/wiki-pi-cilia/gene/view/83544) | [83544](http://severus.dbmi.pitt.edu/wiki-pi-cilia/gene/view/83544) | [0](http://severus.dbmi.pitt.edu/wiki-pi-cilia/search?q=DNAL1) | [3](http://severus.dbmi.pitt.edu/wiki-pi-cilia/search?q=DNAL1) |
| [DNAL4](http://severus.dbmi.pitt.edu/wiki-pi-cilia/gene/view/10126) | [10126](http://severus.dbmi.pitt.edu/wiki-pi-cilia/gene/view/10126) | [9](http://severus.dbmi.pitt.edu/wiki-pi-cilia/search?q=DNAL4) | [9](http://severus.dbmi.pitt.edu/wiki-pi-cilia/search?q=DNAL4) |
| [DNALI1](http://severus.dbmi.pitt.edu/wiki-pi-cilia/gene/view/7802) | [7802](http://severus.dbmi.pitt.edu/wiki-pi-cilia/gene/view/7802) | [5](http://severus.dbmi.pitt.edu/wiki-pi-cilia/search?q=DNALI1) | [4](http://severus.dbmi.pitt.edu/wiki-pi-cilia/search?q=DNALI1) |
| [DRC1](http://severus.dbmi.pitt.edu/wiki-pi-cilia/gene/view/92749) | [92749](http://severus.dbmi.pitt.edu/wiki-pi-cilia/gene/view/92749) | [1](http://severus.dbmi.pitt.edu/wiki-pi-cilia/search?q=DRC1) | [4](http://severus.dbmi.pitt.edu/wiki-pi-cilia/search?q=DRC1) |
| [DRD1](http://severus.dbmi.pitt.edu/wiki-pi-cilia/gene/view/1812) | [1812](http://severus.dbmi.pitt.edu/wiki-pi-cilia/gene/view/1812) | [11](http://severus.dbmi.pitt.edu/wiki-pi-cilia/search?q=DRD1) | [6](http://severus.dbmi.pitt.edu/wiki-pi-cilia/search?q=DRD1) |
| [DRD2](http://severus.dbmi.pitt.edu/wiki-pi-cilia/gene/view/1813) | [1813](http://severus.dbmi.pitt.edu/wiki-pi-cilia/gene/view/1813) | [20](http://severus.dbmi.pitt.edu/wiki-pi-cilia/search?q=DRD2) | [13](http://severus.dbmi.pitt.edu/wiki-pi-cilia/search?q=DRD2) |
| [DRD5](http://severus.dbmi.pitt.edu/wiki-pi-cilia/gene/view/1816) | [1816](http://severus.dbmi.pitt.edu/wiki-pi-cilia/gene/view/1816) | [5](http://severus.dbmi.pitt.edu/wiki-pi-cilia/search?q=DRD5) | [2](http://severus.dbmi.pitt.edu/wiki-pi-cilia/search?q=DRD5) |
| [DYNLL1](http://severus.dbmi.pitt.edu/wiki-pi-cilia/gene/view/8655) | [8655](http://severus.dbmi.pitt.edu/wiki-pi-cilia/gene/view/8655) | [76](http://severus.dbmi.pitt.edu/wiki-pi-cilia/search?q=DYNLL1) | [7](http://severus.dbmi.pitt.edu/wiki-pi-cilia/search?q=DYNLL1) |
| [DYNLL2](http://severus.dbmi.pitt.edu/wiki-pi-cilia/gene/view/140735) | [140735](http://severus.dbmi.pitt.edu/wiki-pi-cilia/gene/view/140735) | [47](http://severus.dbmi.pitt.edu/wiki-pi-cilia/search?q=DYNLL2) | [11](http://severus.dbmi.pitt.edu/wiki-pi-cilia/search?q=DYNLL2) |
| [DYNLRB1](http://severus.dbmi.pitt.edu/wiki-pi-cilia/gene/view/83658) | [83658](http://severus.dbmi.pitt.edu/wiki-pi-cilia/gene/view/83658) | [6](http://severus.dbmi.pitt.edu/wiki-pi-cilia/search?q=DYNLRB1) | [2](http://severus.dbmi.pitt.edu/wiki-pi-cilia/search?q=DYNLRB1) |
| [DYNLRB2](http://severus.dbmi.pitt.edu/wiki-pi-cilia/gene/view/83657) | [83657](http://severus.dbmi.pitt.edu/wiki-pi-cilia/gene/view/83657) | [4](http://severus.dbmi.pitt.edu/wiki-pi-cilia/search?q=DYNLRB2) | [8](http://severus.dbmi.pitt.edu/wiki-pi-cilia/search?q=DYNLRB2) |
| [DYNLT1](http://severus.dbmi.pitt.edu/wiki-pi-cilia/gene/view/6993) | [6993](http://severus.dbmi.pitt.edu/wiki-pi-cilia/gene/view/6993) | [28](http://severus.dbmi.pitt.edu/wiki-pi-cilia/search?q=DYNLT1) | [7](http://severus.dbmi.pitt.edu/wiki-pi-cilia/search?q=DYNLT1) |
| [DYNLT3](http://severus.dbmi.pitt.edu/wiki-pi-cilia/gene/view/6990) | [6990](http://severus.dbmi.pitt.edu/wiki-pi-cilia/gene/view/6990) | [7](http://severus.dbmi.pitt.edu/wiki-pi-cilia/search?q=DYNLT3) | [4](http://severus.dbmi.pitt.edu/wiki-pi-cilia/search?q=DYNLT3) |
| [DYX1C1](http://severus.dbmi.pitt.edu/wiki-pi-cilia/gene/view/161582) | [161582](http://severus.dbmi.pitt.edu/wiki-pi-cilia/gene/view/161582) | [4](http://severus.dbmi.pitt.edu/wiki-pi-cilia/search?q=DYX1C1) | [3](http://severus.dbmi.pitt.edu/wiki-pi-cilia/search?q=DYX1C1) |
| [DZIP1L](http://severus.dbmi.pitt.edu/wiki-pi-cilia/gene/view/199221) | [199221](http://severus.dbmi.pitt.edu/wiki-pi-cilia/gene/view/199221) | [4](http://severus.dbmi.pitt.edu/wiki-pi-cilia/search?q=DZIP1L) | [3](http://severus.dbmi.pitt.edu/wiki-pi-cilia/search?q=DZIP1L) |
| [EFHC1](http://severus.dbmi.pitt.edu/wiki-pi-cilia/gene/view/114327) | [114327](http://severus.dbmi.pitt.edu/wiki-pi-cilia/gene/view/114327) | [6](http://severus.dbmi.pitt.edu/wiki-pi-cilia/search?q=EFHC1) | [2](http://severus.dbmi.pitt.edu/wiki-pi-cilia/search?q=EFHC1) |
| [ESYT3](http://severus.dbmi.pitt.edu/wiki-pi-cilia/gene/view/83850) | [83850](http://severus.dbmi.pitt.edu/wiki-pi-cilia/gene/view/83850) | [0](http://severus.dbmi.pitt.edu/wiki-pi-cilia/search?q=ESYT3) | [2](http://severus.dbmi.pitt.edu/wiki-pi-cilia/search?q=ESYT3) |
| [GALR2](http://severus.dbmi.pitt.edu/wiki-pi-cilia/gene/view/8811) | [8811](http://severus.dbmi.pitt.edu/wiki-pi-cilia/gene/view/8811) | [1](http://severus.dbmi.pitt.edu/wiki-pi-cilia/search?q=GALR2) | [2](http://severus.dbmi.pitt.edu/wiki-pi-cilia/search?q=GALR2) |
| [GALR3](http://severus.dbmi.pitt.edu/wiki-pi-cilia/gene/view/8484) | [8484](http://severus.dbmi.pitt.edu/wiki-pi-cilia/gene/view/8484) | [0](http://severus.dbmi.pitt.edu/wiki-pi-cilia/search?q=GALR3) | [4](http://severus.dbmi.pitt.edu/wiki-pi-cilia/search?q=GALR3) |
| [GAS8](http://severus.dbmi.pitt.edu/wiki-pi-cilia/gene/view/2622) | [2622](http://severus.dbmi.pitt.edu/wiki-pi-cilia/gene/view/2622) | [0](http://severus.dbmi.pitt.edu/wiki-pi-cilia/search?q=GAS8) | [7](http://severus.dbmi.pitt.edu/wiki-pi-cilia/search?q=GAS8) |
| [GPR161](http://severus.dbmi.pitt.edu/wiki-pi-cilia/gene/view/23432) | [23432](http://severus.dbmi.pitt.edu/wiki-pi-cilia/gene/view/23432) | [0](http://severus.dbmi.pitt.edu/wiki-pi-cilia/search?q=GPR161) | [3](http://severus.dbmi.pitt.edu/wiki-pi-cilia/search?q=GPR161) |
| [GPR83](http://severus.dbmi.pitt.edu/wiki-pi-cilia/gene/view/10888) | [10888](http://severus.dbmi.pitt.edu/wiki-pi-cilia/gene/view/10888) | [0](http://severus.dbmi.pitt.edu/wiki-pi-cilia/search?q=GPR83) | [12](http://severus.dbmi.pitt.edu/wiki-pi-cilia/search?q=GPR83) |
| [HEPH](http://severus.dbmi.pitt.edu/wiki-pi-cilia/gene/view/9843) | [9843](http://severus.dbmi.pitt.edu/wiki-pi-cilia/gene/view/9843) | [0](http://severus.dbmi.pitt.edu/wiki-pi-cilia/search?q=HEPH) | [1](http://severus.dbmi.pitt.edu/wiki-pi-cilia/search?q=HEPH) |
| [HSPB11](http://severus.dbmi.pitt.edu/wiki-pi-cilia/gene/view/51668) | [51668](http://severus.dbmi.pitt.edu/wiki-pi-cilia/gene/view/51668) | [2](http://severus.dbmi.pitt.edu/wiki-pi-cilia/search?q=HSPB11) | [4](http://severus.dbmi.pitt.edu/wiki-pi-cilia/search?q=HSPB11) |
| [HYDIN](http://severus.dbmi.pitt.edu/wiki-pi-cilia/gene/view/54768) | [54768](http://severus.dbmi.pitt.edu/wiki-pi-cilia/gene/view/54768) | [1](http://severus.dbmi.pitt.edu/wiki-pi-cilia/search?q=HYDIN) | [2](http://severus.dbmi.pitt.edu/wiki-pi-cilia/search?q=HYDIN) |
| [IFT122](http://severus.dbmi.pitt.edu/wiki-pi-cilia/gene/view/55764) | [55764](http://severus.dbmi.pitt.edu/wiki-pi-cilia/gene/view/55764) | [1](http://severus.dbmi.pitt.edu/wiki-pi-cilia/search?q=IFT122) | [2](http://severus.dbmi.pitt.edu/wiki-pi-cilia/search?q=IFT122) |
| [IFT140](http://severus.dbmi.pitt.edu/wiki-pi-cilia/gene/view/9742) | [9742](http://severus.dbmi.pitt.edu/wiki-pi-cilia/gene/view/9742) | [4](http://severus.dbmi.pitt.edu/wiki-pi-cilia/search?q=IFT140) | [7](http://severus.dbmi.pitt.edu/wiki-pi-cilia/search?q=IFT140) |
| [IFT172](http://severus.dbmi.pitt.edu/wiki-pi-cilia/gene/view/26160) | [26160](http://severus.dbmi.pitt.edu/wiki-pi-cilia/gene/view/26160) | [2](http://severus.dbmi.pitt.edu/wiki-pi-cilia/search?q=IFT172) | [4](http://severus.dbmi.pitt.edu/wiki-pi-cilia/search?q=IFT172) |
| [IFT20](http://severus.dbmi.pitt.edu/wiki-pi-cilia/gene/view/90410) | [90410](http://severus.dbmi.pitt.edu/wiki-pi-cilia/gene/view/90410) | [28](http://severus.dbmi.pitt.edu/wiki-pi-cilia/search?q=IFT20) | [8](http://severus.dbmi.pitt.edu/wiki-pi-cilia/search?q=IFT20) |
| [IFT27](http://severus.dbmi.pitt.edu/wiki-pi-cilia/gene/view/11020) | [11020](http://severus.dbmi.pitt.edu/wiki-pi-cilia/gene/view/11020) | [2](http://severus.dbmi.pitt.edu/wiki-pi-cilia/search?q=IFT27) | [5](http://severus.dbmi.pitt.edu/wiki-pi-cilia/search?q=IFT27) |
| [IFT43](http://severus.dbmi.pitt.edu/wiki-pi-cilia/gene/view/112752) | [112752](http://severus.dbmi.pitt.edu/wiki-pi-cilia/gene/view/112752) | [2](http://severus.dbmi.pitt.edu/wiki-pi-cilia/search?q=IFT43) | [3](http://severus.dbmi.pitt.edu/wiki-pi-cilia/search?q=IFT43) |
| [IFT46](http://severus.dbmi.pitt.edu/wiki-pi-cilia/gene/view/56912) | [56912](http://severus.dbmi.pitt.edu/wiki-pi-cilia/gene/view/56912) | [0](http://severus.dbmi.pitt.edu/wiki-pi-cilia/search?q=IFT46) | [6](http://severus.dbmi.pitt.edu/wiki-pi-cilia/search?q=IFT46) |
| [IFT52](http://severus.dbmi.pitt.edu/wiki-pi-cilia/gene/view/51098) | [51098](http://severus.dbmi.pitt.edu/wiki-pi-cilia/gene/view/51098) | [1](http://severus.dbmi.pitt.edu/wiki-pi-cilia/search?q=IFT52) | [2](http://severus.dbmi.pitt.edu/wiki-pi-cilia/search?q=IFT52) |
| [IFT57](http://severus.dbmi.pitt.edu/wiki-pi-cilia/gene/view/55081) | [55081](http://severus.dbmi.pitt.edu/wiki-pi-cilia/gene/view/55081) | [4](http://severus.dbmi.pitt.edu/wiki-pi-cilia/search?q=IFT57) | [6](http://severus.dbmi.pitt.edu/wiki-pi-cilia/search?q=IFT57) |
| [IFT74](http://severus.dbmi.pitt.edu/wiki-pi-cilia/gene/view/80173) | [80173](http://severus.dbmi.pitt.edu/wiki-pi-cilia/gene/view/80173) | [0](http://severus.dbmi.pitt.edu/wiki-pi-cilia/search?q=IFT74) | [0](http://severus.dbmi.pitt.edu/wiki-pi-cilia/search?q=IFT74) |
| [IFT80](http://severus.dbmi.pitt.edu/wiki-pi-cilia/gene/view/57560) | [57560](http://severus.dbmi.pitt.edu/wiki-pi-cilia/gene/view/57560) | [1](http://severus.dbmi.pitt.edu/wiki-pi-cilia/search?q=IFT80) | [5](http://severus.dbmi.pitt.edu/wiki-pi-cilia/search?q=IFT80) |
| [IFT81](http://severus.dbmi.pitt.edu/wiki-pi-cilia/gene/view/28981) | [28981](http://severus.dbmi.pitt.edu/wiki-pi-cilia/gene/view/28981) | [1](http://severus.dbmi.pitt.edu/wiki-pi-cilia/search?q=IFT81) | [6](http://severus.dbmi.pitt.edu/wiki-pi-cilia/search?q=IFT81) |
| [IFT88](http://severus.dbmi.pitt.edu/wiki-pi-cilia/gene/view/8100) | [8100](http://severus.dbmi.pitt.edu/wiki-pi-cilia/gene/view/8100) | [6](http://severus.dbmi.pitt.edu/wiki-pi-cilia/search?q=IFT88) | [6](http://severus.dbmi.pitt.edu/wiki-pi-cilia/search?q=IFT88) |
| [INVS](http://severus.dbmi.pitt.edu/wiki-pi-cilia/gene/view/27130) | [27130](http://severus.dbmi.pitt.edu/wiki-pi-cilia/gene/view/27130) | [5](http://severus.dbmi.pitt.edu/wiki-pi-cilia/search?q=INVS) | [2](http://severus.dbmi.pitt.edu/wiki-pi-cilia/search?q=INVS) |
| [IQCB1](http://severus.dbmi.pitt.edu/wiki-pi-cilia/gene/view/9657) | [9657](http://severus.dbmi.pitt.edu/wiki-pi-cilia/gene/view/9657) | [1](http://severus.dbmi.pitt.edu/wiki-pi-cilia/search?q=IQCB1) | [6](http://severus.dbmi.pitt.edu/wiki-pi-cilia/search?q=IQCB1) |
| [LCA5](http://severus.dbmi.pitt.edu/wiki-pi-cilia/gene/view/167691) | [167691](http://severus.dbmi.pitt.edu/wiki-pi-cilia/gene/view/167691) | [3](http://severus.dbmi.pitt.edu/wiki-pi-cilia/search?q=LCA5) | [1](http://severus.dbmi.pitt.edu/wiki-pi-cilia/search?q=LCA5) |
| [LOXHD1](http://severus.dbmi.pitt.edu/wiki-pi-cilia/gene/view/125336) | [125336](http://severus.dbmi.pitt.edu/wiki-pi-cilia/gene/view/125336) | [0](http://severus.dbmi.pitt.edu/wiki-pi-cilia/search?q=LOXHD1) | [4](http://severus.dbmi.pitt.edu/wiki-pi-cilia/search?q=LOXHD1) |
| [LRRC48](http://severus.dbmi.pitt.edu/wiki-pi-cilia/gene/view/83450) | [83450](http://severus.dbmi.pitt.edu/wiki-pi-cilia/gene/view/83450) | [0](http://severus.dbmi.pitt.edu/wiki-pi-cilia/search?q=LRRC48) | [10](http://severus.dbmi.pitt.edu/wiki-pi-cilia/search?q=LRRC48) |
| [LRRC56](http://severus.dbmi.pitt.edu/wiki-pi-cilia/gene/view/115399) | [115399](http://severus.dbmi.pitt.edu/wiki-pi-cilia/gene/view/115399) | [0](http://severus.dbmi.pitt.edu/wiki-pi-cilia/search?q=LRRC56) | [2](http://severus.dbmi.pitt.edu/wiki-pi-cilia/search?q=LRRC56) |
| [LRRC6](http://severus.dbmi.pitt.edu/wiki-pi-cilia/gene/view/23639) | [23639](http://severus.dbmi.pitt.edu/wiki-pi-cilia/gene/view/23639) | [2](http://severus.dbmi.pitt.edu/wiki-pi-cilia/search?q=LRRC6) | [1](http://severus.dbmi.pitt.edu/wiki-pi-cilia/search?q=LRRC6) |
| [MCHR1](http://severus.dbmi.pitt.edu/wiki-pi-cilia/gene/view/2847) | [2847](http://severus.dbmi.pitt.edu/wiki-pi-cilia/gene/view/2847) | [2](http://severus.dbmi.pitt.edu/wiki-pi-cilia/search?q=MCHR1) | [8](http://severus.dbmi.pitt.edu/wiki-pi-cilia/search?q=MCHR1) |
| [MKS1](http://severus.dbmi.pitt.edu/wiki-pi-cilia/gene/view/54903) | [54903](http://severus.dbmi.pitt.edu/wiki-pi-cilia/gene/view/54903) | [2](http://severus.dbmi.pitt.edu/wiki-pi-cilia/search?q=MKS1) | [5](http://severus.dbmi.pitt.edu/wiki-pi-cilia/search?q=MKS1) |
| [NEK8](http://severus.dbmi.pitt.edu/wiki-pi-cilia/gene/view/284086) | [284086](http://severus.dbmi.pitt.edu/wiki-pi-cilia/gene/view/284086) | [4](http://severus.dbmi.pitt.edu/wiki-pi-cilia/search?q=NEK8) | [2](http://severus.dbmi.pitt.edu/wiki-pi-cilia/search?q=NEK8) |
| [NME5](http://severus.dbmi.pitt.edu/wiki-pi-cilia/gene/view/8382) | [8382](http://severus.dbmi.pitt.edu/wiki-pi-cilia/gene/view/8382) | [2](http://severus.dbmi.pitt.edu/wiki-pi-cilia/search?q=NME5) | [8](http://severus.dbmi.pitt.edu/wiki-pi-cilia/search?q=NME5) |
| [NME8](http://severus.dbmi.pitt.edu/wiki-pi-cilia/gene/view/51314) | [51314](http://severus.dbmi.pitt.edu/wiki-pi-cilia/gene/view/51314) | [1](http://severus.dbmi.pitt.edu/wiki-pi-cilia/search?q=NME8) | [2](http://severus.dbmi.pitt.edu/wiki-pi-cilia/search?q=NME8) |
| [NPHP1](http://severus.dbmi.pitt.edu/wiki-pi-cilia/gene/view/4867) | [4867](http://severus.dbmi.pitt.edu/wiki-pi-cilia/gene/view/4867) | [15](http://severus.dbmi.pitt.edu/wiki-pi-cilia/search?q=NPHP1) | [3](http://severus.dbmi.pitt.edu/wiki-pi-cilia/search?q=NPHP1) |
| [NPHP3](http://severus.dbmi.pitt.edu/wiki-pi-cilia/gene/view/27031) | [27031](http://severus.dbmi.pitt.edu/wiki-pi-cilia/gene/view/27031) | [2](http://severus.dbmi.pitt.edu/wiki-pi-cilia/search?q=NPHP3) | [15](http://severus.dbmi.pitt.edu/wiki-pi-cilia/search?q=NPHP3) |
| [NPHP4](http://severus.dbmi.pitt.edu/wiki-pi-cilia/gene/view/261734) | [261734](http://severus.dbmi.pitt.edu/wiki-pi-cilia/gene/view/261734) | [1](http://severus.dbmi.pitt.edu/wiki-pi-cilia/search?q=NPHP4) | [8](http://severus.dbmi.pitt.edu/wiki-pi-cilia/search?q=NPHP4) |
| [NPY2R](http://severus.dbmi.pitt.edu/wiki-pi-cilia/gene/view/4887) | [4887](http://severus.dbmi.pitt.edu/wiki-pi-cilia/gene/view/4887) | [4](http://severus.dbmi.pitt.edu/wiki-pi-cilia/search?q=NPY2R) | [3](http://severus.dbmi.pitt.edu/wiki-pi-cilia/search?q=NPY2R) |
| [NPY5R](http://severus.dbmi.pitt.edu/wiki-pi-cilia/gene/view/4889) | [4889](http://severus.dbmi.pitt.edu/wiki-pi-cilia/gene/view/4889) | [4](http://severus.dbmi.pitt.edu/wiki-pi-cilia/search?q=NPY5R) | [2](http://severus.dbmi.pitt.edu/wiki-pi-cilia/search?q=NPY5R) |
| [PDCD6IP](http://severus.dbmi.pitt.edu/wiki-pi-cilia/gene/view/10015) | [10015](http://severus.dbmi.pitt.edu/wiki-pi-cilia/gene/view/10015) | [37](http://severus.dbmi.pitt.edu/wiki-pi-cilia/search?q=PDCD6IP) | [7](http://severus.dbmi.pitt.edu/wiki-pi-cilia/search?q=PDCD6IP) |
| [PIH1D1](http://severus.dbmi.pitt.edu/wiki-pi-cilia/gene/view/55011) | [55011](http://severus.dbmi.pitt.edu/wiki-pi-cilia/gene/view/55011) | [31](http://severus.dbmi.pitt.edu/wiki-pi-cilia/search?q=PIH1D1) | [5](http://severus.dbmi.pitt.edu/wiki-pi-cilia/search?q=PIH1D1) |
| [PKD1](http://severus.dbmi.pitt.edu/wiki-pi-cilia/gene/view/5310) | [5310](http://severus.dbmi.pitt.edu/wiki-pi-cilia/gene/view/5310) | [24](http://severus.dbmi.pitt.edu/wiki-pi-cilia/search?q=PKD1) | [10](http://severus.dbmi.pitt.edu/wiki-pi-cilia/search?q=PKD1) |
| [PKD1L1](http://severus.dbmi.pitt.edu/wiki-pi-cilia/gene/view/168507) | [168507](http://severus.dbmi.pitt.edu/wiki-pi-cilia/gene/view/168507) | [0](http://severus.dbmi.pitt.edu/wiki-pi-cilia/search?q=PKD1L1) | [10](http://severus.dbmi.pitt.edu/wiki-pi-cilia/search?q=PKD1L1) |
| [PKD2](http://severus.dbmi.pitt.edu/wiki-pi-cilia/gene/view/5311) | [5311](http://severus.dbmi.pitt.edu/wiki-pi-cilia/gene/view/5311) | [14](http://severus.dbmi.pitt.edu/wiki-pi-cilia/search?q=PKD2) | [8](http://severus.dbmi.pitt.edu/wiki-pi-cilia/search?q=PKD2) |
| [PKD2L1](http://severus.dbmi.pitt.edu/wiki-pi-cilia/gene/view/9033) | [9033](http://severus.dbmi.pitt.edu/wiki-pi-cilia/gene/view/9033) | [3](http://severus.dbmi.pitt.edu/wiki-pi-cilia/search?q=PKD2L1) | [6](http://severus.dbmi.pitt.edu/wiki-pi-cilia/search?q=PKD2L1) |
| [PKHD1](http://severus.dbmi.pitt.edu/wiki-pi-cilia/gene/view/5314) | [5314](http://severus.dbmi.pitt.edu/wiki-pi-cilia/gene/view/5314) | [1](http://severus.dbmi.pitt.edu/wiki-pi-cilia/search?q=PKHD1) | [7](http://severus.dbmi.pitt.edu/wiki-pi-cilia/search?q=PKHD1) |
| [PPIL6](http://severus.dbmi.pitt.edu/wiki-pi-cilia/gene/view/285755) | [285755](http://severus.dbmi.pitt.edu/wiki-pi-cilia/gene/view/285755) | [0](http://severus.dbmi.pitt.edu/wiki-pi-cilia/search?q=PPIL6) | [2](http://severus.dbmi.pitt.edu/wiki-pi-cilia/search?q=PPIL6) |
| [PPP1CB](http://severus.dbmi.pitt.edu/wiki-pi-cilia/gene/view/5500) | [5500](http://severus.dbmi.pitt.edu/wiki-pi-cilia/gene/view/5500) | [26](http://severus.dbmi.pitt.edu/wiki-pi-cilia/search?q=PPP1CB) | [10](http://severus.dbmi.pitt.edu/wiki-pi-cilia/search?q=PPP1CB) |
| [PTCH1](http://severus.dbmi.pitt.edu/wiki-pi-cilia/gene/view/5727) | [5727](http://severus.dbmi.pitt.edu/wiki-pi-cilia/gene/view/5727) | [65](http://severus.dbmi.pitt.edu/wiki-pi-cilia/search?q=PTCH1) | [5](http://severus.dbmi.pitt.edu/wiki-pi-cilia/search?q=PTCH1) |
| [QRFPR](http://severus.dbmi.pitt.edu/wiki-pi-cilia/gene/view/84109) | [84109](http://severus.dbmi.pitt.edu/wiki-pi-cilia/gene/view/84109) | [0](http://severus.dbmi.pitt.edu/wiki-pi-cilia/search?q=QRFPR) | [9](http://severus.dbmi.pitt.edu/wiki-pi-cilia/search?q=QRFPR) |
| [RABL5](http://severus.dbmi.pitt.edu/wiki-pi-cilia/gene/view/64792) | [64792](http://severus.dbmi.pitt.edu/wiki-pi-cilia/gene/view/64792) | [0](http://severus.dbmi.pitt.edu/wiki-pi-cilia/search?q=RABL5) | [0](http://severus.dbmi.pitt.edu/wiki-pi-cilia/search?q=RABL5) |
| [ROPN1L](http://severus.dbmi.pitt.edu/wiki-pi-cilia/gene/view/83853) | [83853](http://severus.dbmi.pitt.edu/wiki-pi-cilia/gene/view/83853) | [3](http://severus.dbmi.pitt.edu/wiki-pi-cilia/search?q=ROPN1L) | [4](http://severus.dbmi.pitt.edu/wiki-pi-cilia/search?q=ROPN1L) |
| [RPGR](http://severus.dbmi.pitt.edu/wiki-pi-cilia/gene/view/6103) | [6103](http://severus.dbmi.pitt.edu/wiki-pi-cilia/gene/view/6103) | [11](http://severus.dbmi.pitt.edu/wiki-pi-cilia/search?q=RPGR) | [7](http://severus.dbmi.pitt.edu/wiki-pi-cilia/search?q=RPGR) |
| [RPGRIP1](http://severus.dbmi.pitt.edu/wiki-pi-cilia/gene/view/57096) | [57096](http://severus.dbmi.pitt.edu/wiki-pi-cilia/gene/view/57096) | [23](http://severus.dbmi.pitt.edu/wiki-pi-cilia/search?q=RPGRIP1) | [1](http://severus.dbmi.pitt.edu/wiki-pi-cilia/search?q=RPGRIP1) |
| [RPGRIP1L](http://severus.dbmi.pitt.edu/wiki-pi-cilia/gene/view/23322) | [23322](http://severus.dbmi.pitt.edu/wiki-pi-cilia/gene/view/23322) | [3](http://severus.dbmi.pitt.edu/wiki-pi-cilia/search?q=RPGRIP1L) | [2](http://severus.dbmi.pitt.edu/wiki-pi-cilia/search?q=RPGRIP1L) |
| [RSPH1](http://severus.dbmi.pitt.edu/wiki-pi-cilia/gene/view/89765) | [89765](http://severus.dbmi.pitt.edu/wiki-pi-cilia/gene/view/89765) | [1](http://severus.dbmi.pitt.edu/wiki-pi-cilia/search?q=RSPH1) | [7](http://severus.dbmi.pitt.edu/wiki-pi-cilia/search?q=RSPH1) |
| [RSPH10B](http://severus.dbmi.pitt.edu/wiki-pi-cilia/gene/view/222967) | [222967](http://severus.dbmi.pitt.edu/wiki-pi-cilia/gene/view/222967) | [0](http://severus.dbmi.pitt.edu/wiki-pi-cilia/search?q=RSPH10B) | [7](http://severus.dbmi.pitt.edu/wiki-pi-cilia/search?q=RSPH10B) |
| [RSPH3](http://severus.dbmi.pitt.edu/wiki-pi-cilia/gene/view/83861) | [83861](http://severus.dbmi.pitt.edu/wiki-pi-cilia/gene/view/83861) | [5](http://severus.dbmi.pitt.edu/wiki-pi-cilia/search?q=RSPH3) | [6](http://severus.dbmi.pitt.edu/wiki-pi-cilia/search?q=RSPH3) |
| [RSPH4A](http://severus.dbmi.pitt.edu/wiki-pi-cilia/gene/view/345895) | [345895](http://severus.dbmi.pitt.edu/wiki-pi-cilia/gene/view/345895) | [0](http://severus.dbmi.pitt.edu/wiki-pi-cilia/search?q=RSPH4A) | [6](http://severus.dbmi.pitt.edu/wiki-pi-cilia/search?q=RSPH4A) |
| [RSPH6A](http://severus.dbmi.pitt.edu/wiki-pi-cilia/gene/view/81492) | [81492](http://severus.dbmi.pitt.edu/wiki-pi-cilia/gene/view/81492) | [0](http://severus.dbmi.pitt.edu/wiki-pi-cilia/search?q=RSPH6A) | [1](http://severus.dbmi.pitt.edu/wiki-pi-cilia/search?q=RSPH6A) |
| [RSPH9](http://severus.dbmi.pitt.edu/wiki-pi-cilia/gene/view/221421) | [221421](http://severus.dbmi.pitt.edu/wiki-pi-cilia/gene/view/221421) | [2](http://severus.dbmi.pitt.edu/wiki-pi-cilia/search?q=RSPH9) | [9](http://severus.dbmi.pitt.edu/wiki-pi-cilia/search?q=RSPH9) |
| [RTDR1](http://severus.dbmi.pitt.edu/wiki-pi-cilia/gene/view/27156) | [27156](http://severus.dbmi.pitt.edu/wiki-pi-cilia/gene/view/27156) | [13](http://severus.dbmi.pitt.edu/wiki-pi-cilia/search?q=RTDR1) | [0](http://severus.dbmi.pitt.edu/wiki-pi-cilia/search?q=RTDR1) |
| [SMO](http://severus.dbmi.pitt.edu/wiki-pi-cilia/gene/view/6608) | [6608](http://severus.dbmi.pitt.edu/wiki-pi-cilia/gene/view/6608) | [8](http://severus.dbmi.pitt.edu/wiki-pi-cilia/search?q=SMO) | [1](http://severus.dbmi.pitt.edu/wiki-pi-cilia/search?q=SMO) |
| [SPAG1](http://severus.dbmi.pitt.edu/wiki-pi-cilia/gene/view/6674) | [6674](http://severus.dbmi.pitt.edu/wiki-pi-cilia/gene/view/6674) | [3](http://severus.dbmi.pitt.edu/wiki-pi-cilia/search?q=SPAG1) | [7](http://severus.dbmi.pitt.edu/wiki-pi-cilia/search?q=SPAG1) |
| [SPAG16](http://severus.dbmi.pitt.edu/wiki-pi-cilia/gene/view/79582) | [79582](http://severus.dbmi.pitt.edu/wiki-pi-cilia/gene/view/79582) | [2](http://severus.dbmi.pitt.edu/wiki-pi-cilia/search?q=SPAG16) | [1](http://severus.dbmi.pitt.edu/wiki-pi-cilia/search?q=SPAG16) |
| [SPAG17](http://severus.dbmi.pitt.edu/wiki-pi-cilia/gene/view/200162) | [200162](http://severus.dbmi.pitt.edu/wiki-pi-cilia/gene/view/200162) | [0](http://severus.dbmi.pitt.edu/wiki-pi-cilia/search?q=SPAG17) | [2](http://severus.dbmi.pitt.edu/wiki-pi-cilia/search?q=SPAG17) |
| [SPAG6](http://severus.dbmi.pitt.edu/wiki-pi-cilia/gene/view/9576) | [9576](http://severus.dbmi.pitt.edu/wiki-pi-cilia/gene/view/9576) | [2](http://severus.dbmi.pitt.edu/wiki-pi-cilia/search?q=SPAG6) | [2](http://severus.dbmi.pitt.edu/wiki-pi-cilia/search?q=SPAG6) |
| [SPEF2](http://severus.dbmi.pitt.edu/wiki-pi-cilia/gene/view/79925) | [79925](http://severus.dbmi.pitt.edu/wiki-pi-cilia/gene/view/79925) | [0](http://severus.dbmi.pitt.edu/wiki-pi-cilia/search?q=SPEF2) | [10](http://severus.dbmi.pitt.edu/wiki-pi-cilia/search?q=SPEF2) |
| [TCTE1](http://severus.dbmi.pitt.edu/wiki-pi-cilia/gene/view/202500) | [202500](http://severus.dbmi.pitt.edu/wiki-pi-cilia/gene/view/202500) | [1](http://severus.dbmi.pitt.edu/wiki-pi-cilia/search?q=TCTE1) | [7](http://severus.dbmi.pitt.edu/wiki-pi-cilia/search?q=TCTE1) |
| [TCTE3](http://severus.dbmi.pitt.edu/wiki-pi-cilia/gene/view/6991) | [6991](http://severus.dbmi.pitt.edu/wiki-pi-cilia/gene/view/6991) | [1](http://severus.dbmi.pitt.edu/wiki-pi-cilia/search?q=TCTE3) | [4](http://severus.dbmi.pitt.edu/wiki-pi-cilia/search?q=TCTE3) |
| [TCTN1](http://severus.dbmi.pitt.edu/wiki-pi-cilia/gene/view/79600) | [79600](http://severus.dbmi.pitt.edu/wiki-pi-cilia/gene/view/79600) | [0](http://severus.dbmi.pitt.edu/wiki-pi-cilia/search?q=TCTN1) | [3](http://severus.dbmi.pitt.edu/wiki-pi-cilia/search?q=TCTN1) |
| [TCTN2](http://severus.dbmi.pitt.edu/wiki-pi-cilia/gene/view/79867) | [79867](http://severus.dbmi.pitt.edu/wiki-pi-cilia/gene/view/79867) | [0](http://severus.dbmi.pitt.edu/wiki-pi-cilia/search?q=TCTN2) | [3](http://severus.dbmi.pitt.edu/wiki-pi-cilia/search?q=TCTN2) |
| [TCTN3](http://severus.dbmi.pitt.edu/wiki-pi-cilia/gene/view/26123) | [26123](http://severus.dbmi.pitt.edu/wiki-pi-cilia/gene/view/26123) | [0](http://severus.dbmi.pitt.edu/wiki-pi-cilia/search?q=TCTN3) | [0](http://severus.dbmi.pitt.edu/wiki-pi-cilia/search?q=TCTN3) |
| [TMEM17](http://severus.dbmi.pitt.edu/wiki-pi-cilia/gene/view/200728) | [200728](http://severus.dbmi.pitt.edu/wiki-pi-cilia/gene/view/200728) | [0](http://severus.dbmi.pitt.edu/wiki-pi-cilia/search?q=TMEM17) | [3](http://severus.dbmi.pitt.edu/wiki-pi-cilia/search?q=TMEM17) |
| [TMEM216](http://severus.dbmi.pitt.edu/wiki-pi-cilia/gene/view/51259) | [51259](http://severus.dbmi.pitt.edu/wiki-pi-cilia/gene/view/51259) | [1](http://severus.dbmi.pitt.edu/wiki-pi-cilia/search?q=TMEM216) | [9](http://severus.dbmi.pitt.edu/wiki-pi-cilia/search?q=TMEM216) |
| [TMEM231](http://severus.dbmi.pitt.edu/wiki-pi-cilia/gene/view/79583) | [79583](http://severus.dbmi.pitt.edu/wiki-pi-cilia/gene/view/79583) | [3](http://severus.dbmi.pitt.edu/wiki-pi-cilia/search?q=TMEM231) | [4](http://severus.dbmi.pitt.edu/wiki-pi-cilia/search?q=TMEM231) |
| [TMEM237](http://severus.dbmi.pitt.edu/wiki-pi-cilia/gene/view/65062) | [65062](http://severus.dbmi.pitt.edu/wiki-pi-cilia/gene/view/65062) | [2](http://severus.dbmi.pitt.edu/wiki-pi-cilia/search?q=TMEM237) | [2](http://severus.dbmi.pitt.edu/wiki-pi-cilia/search?q=TMEM237) |
| [TMEM30B](http://severus.dbmi.pitt.edu/wiki-pi-cilia/gene/view/161291) | [161291](http://severus.dbmi.pitt.edu/wiki-pi-cilia/gene/view/161291) | [0](http://severus.dbmi.pitt.edu/wiki-pi-cilia/search?q=TMEM30B) | [4](http://severus.dbmi.pitt.edu/wiki-pi-cilia/search?q=TMEM30B) |
| [TMEM67](http://severus.dbmi.pitt.edu/wiki-pi-cilia/gene/view/91147) | [91147](http://severus.dbmi.pitt.edu/wiki-pi-cilia/gene/view/91147) | [1](http://severus.dbmi.pitt.edu/wiki-pi-cilia/search?q=TMEM67) | [4](http://severus.dbmi.pitt.edu/wiki-pi-cilia/search?q=TMEM67) |
| [TRAF3IP1](http://severus.dbmi.pitt.edu/wiki-pi-cilia/gene/view/26146) | [26146](http://severus.dbmi.pitt.edu/wiki-pi-cilia/gene/view/26146) | [27](http://severus.dbmi.pitt.edu/wiki-pi-cilia/search?q=TRAF3IP1) | [3](http://severus.dbmi.pitt.edu/wiki-pi-cilia/search?q=TRAF3IP1) |
| [TSG101](http://severus.dbmi.pitt.edu/wiki-pi-cilia/gene/view/7251) | [7251](http://severus.dbmi.pitt.edu/wiki-pi-cilia/gene/view/7251) | [86](http://severus.dbmi.pitt.edu/wiki-pi-cilia/search?q=TSG101) | [13](http://severus.dbmi.pitt.edu/wiki-pi-cilia/search?q=TSG101) |
| [TTC21B](http://severus.dbmi.pitt.edu/wiki-pi-cilia/gene/view/79809) | [79809](http://severus.dbmi.pitt.edu/wiki-pi-cilia/gene/view/79809) | [0](http://severus.dbmi.pitt.edu/wiki-pi-cilia/search?q=TTC21B) | [4](http://severus.dbmi.pitt.edu/wiki-pi-cilia/search?q=TTC21B) |
| [TTC28](http://severus.dbmi.pitt.edu/wiki-pi-cilia/gene/view/23331) | [23331](http://severus.dbmi.pitt.edu/wiki-pi-cilia/gene/view/23331) | [3](http://severus.dbmi.pitt.edu/wiki-pi-cilia/search?q=TTC28) | [4](http://severus.dbmi.pitt.edu/wiki-pi-cilia/search?q=TTC28) |
| [TTC30B](http://severus.dbmi.pitt.edu/wiki-pi-cilia/gene/view/150737) | [150737](http://severus.dbmi.pitt.edu/wiki-pi-cilia/gene/view/150737) | [0](http://severus.dbmi.pitt.edu/wiki-pi-cilia/search?q=TTC30B) | [7](http://severus.dbmi.pitt.edu/wiki-pi-cilia/search?q=TTC30B) |
| [TTC8](http://severus.dbmi.pitt.edu/wiki-pi-cilia/gene/view/123016) | [123016](http://severus.dbmi.pitt.edu/wiki-pi-cilia/gene/view/123016) | [1](http://severus.dbmi.pitt.edu/wiki-pi-cilia/search?q=TTC8) | [1](http://severus.dbmi.pitt.edu/wiki-pi-cilia/search?q=TTC8) |
| [VPS28](http://severus.dbmi.pitt.edu/wiki-pi-cilia/gene/view/51160) | [51160](http://severus.dbmi.pitt.edu/wiki-pi-cilia/gene/view/51160) | [27](http://severus.dbmi.pitt.edu/wiki-pi-cilia/search?q=VPS28) | [7](http://severus.dbmi.pitt.edu/wiki-pi-cilia/search?q=VPS28) |
| [VPS37A](http://severus.dbmi.pitt.edu/wiki-pi-cilia/gene/view/137492) | [137492](http://severus.dbmi.pitt.edu/wiki-pi-cilia/gene/view/137492) | [7](http://severus.dbmi.pitt.edu/wiki-pi-cilia/search?q=VPS37A) | [4](http://severus.dbmi.pitt.edu/wiki-pi-cilia/search?q=VPS37A) |
| [VPS4B](http://severus.dbmi.pitt.edu/wiki-pi-cilia/gene/view/9525) | [9525](http://severus.dbmi.pitt.edu/wiki-pi-cilia/gene/view/9525) | [7](http://severus.dbmi.pitt.edu/wiki-pi-cilia/search?q=VPS4B) | [3](http://severus.dbmi.pitt.edu/wiki-pi-cilia/search?q=VPS4B) |
| [WDR19](http://severus.dbmi.pitt.edu/wiki-pi-cilia/gene/view/57728) | [57728](http://severus.dbmi.pitt.edu/wiki-pi-cilia/gene/view/57728) | [0](http://severus.dbmi.pitt.edu/wiki-pi-cilia/search?q=WDR19) | [3](http://severus.dbmi.pitt.edu/wiki-pi-cilia/search?q=WDR19) |
| [WDR35](http://severus.dbmi.pitt.edu/wiki-pi-cilia/gene/view/57539) | [57539](http://severus.dbmi.pitt.edu/wiki-pi-cilia/gene/view/57539) | [0](http://severus.dbmi.pitt.edu/wiki-pi-cilia/search?q=WDR35) | [0](http://severus.dbmi.pitt.edu/wiki-pi-cilia/search?q=WDR35) |
| [WDR63](http://severus.dbmi.pitt.edu/wiki-pi-cilia/gene/view/126820) | [126820](http://severus.dbmi.pitt.edu/wiki-pi-cilia/gene/view/126820) | [0](http://severus.dbmi.pitt.edu/wiki-pi-cilia/search?q=WDR63) | [2](http://severus.dbmi.pitt.edu/wiki-pi-cilia/search?q=WDR63) |
| [WDR78](http://severus.dbmi.pitt.edu/wiki-pi-cilia/gene/view/79819) | [79819](http://severus.dbmi.pitt.edu/wiki-pi-cilia/gene/view/79819) | [0](http://severus.dbmi.pitt.edu/wiki-pi-cilia/search?q=WDR78) | [1](http://severus.dbmi.pitt.edu/wiki-pi-cilia/search?q=WDR78) |
| [ZMYND10](http://severus.dbmi.pitt.edu/wiki-pi-cilia/gene/view/51364) | [51364](http://severus.dbmi.pitt.edu/wiki-pi-cilia/gene/view/51364) | [10](http://severus.dbmi.pitt.edu/wiki-pi-cilia/search?q=ZMYND10) | [2](http://severus.dbmi.pitt.edu/wiki-pi-cilia/search?q=ZMYND10) |
| [ZMYND12](http://severus.dbmi.pitt.edu/wiki-pi-cilia/gene/view/84217) | [84217](http://severus.dbmi.pitt.edu/wiki-pi-cilia/gene/view/84217) | [0](http://severus.dbmi.pitt.edu/wiki-pi-cilia/search?q=ZMYND12) | [3](http://severus.dbmi.pitt.edu/wiki-pi-cilia/search?q=ZMYND12) |

1. Contributed equally [↑](#footnote-ref-1)
