# Supplementary material for: Cilia interactome with predicted protein–protein interactions reveals connections to Alzheimer’s disease, aging and other neuropsychiatric processes
Source: Sci Rep. 2020 Sep 24;10:15629. doi: 10.1038/s41598-020-72024-4 (PMC7515907; doi:10.1038/s41598-020-72024-4)
Supplement: Supplementary file 5 — Supplementary Information 5. [file 41598_2020_72024_MOESM5_ESM.docx]

**Cilia interactome with predicted protein-protein interactions reveals connections to Alzheimer’s disease, Aging and other Neuropsychiatric Processes**

Kalyani B. Karunakaran^1^^[[1]](#footnote-1)^, Srilakshmi Chaparala^2,3†^, Cecilia W. Lo^4^ and Madhavi K. Ganapathiraju^2,5^*

^1^Indian Institute of Science, Bangalore, India

^2^Department of Biomedical Informatics, ^3^Health Sciences Library System, and ^4^Department of Developmental Biology, School of Medicine, and ^5^Intelligent Systems Program, School of Computing and Information, University of Pittsburgh, Pittsburgh, PA, USA

*To whom correspondence should be addressed: Madhavi K. Ganapathiraju, Department of Biomedical Informatics, University of Pittsburgh, Pittsburgh, PA 15206, USA. madhavi@pitt.edu


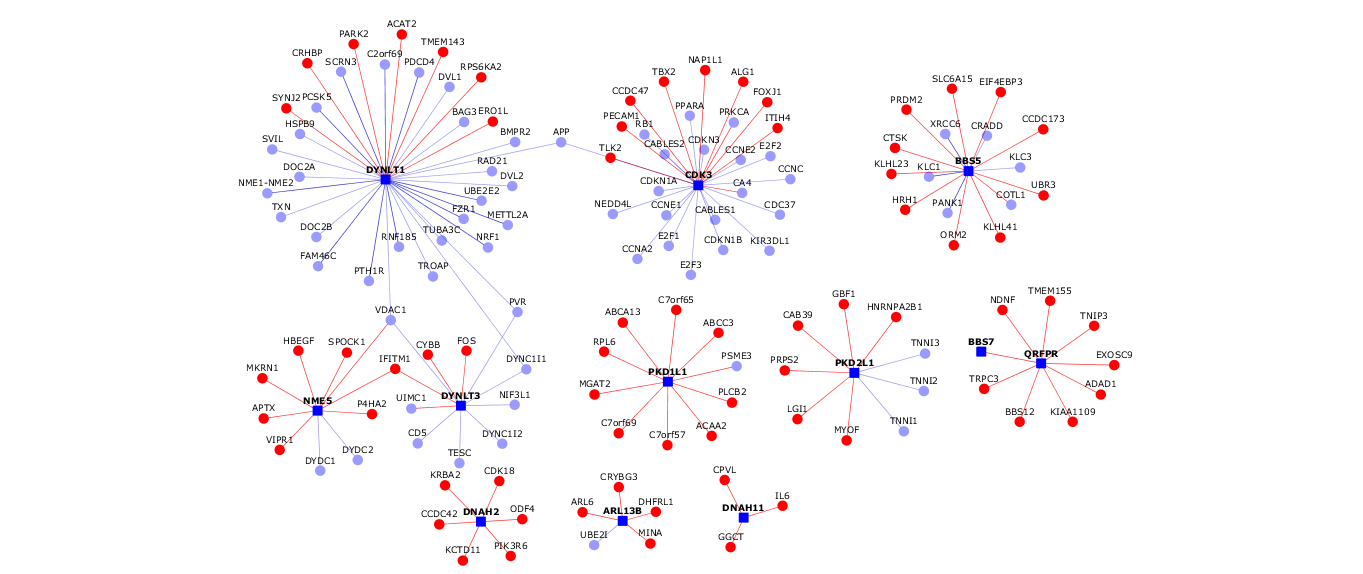


**Figure. Some novel PPIs in the Cilia interactome.** Cilia genes are shown as small dark-blue colored nodes and interactor genes are larger round nodes; the interactors are colored in light blue if they are previously known interactors and in red if they are found only through novel PPIs. PPIs are shown as edges, where blue color edges are known PPIs and red color edges are novel predicted PPIs.

## Novel PPIs involved in neuropsychiatric disorders

**LGI1**, associated with temporal lobe epilepsy (seizure disorder), has been predicted as a novel interactor of the ciliary protein **PKD2L1** (figure) (1). **PKD2L1** (*polycystic kidney disease 2-like 1 protein*), which is implicated in epilepsy, controls excitability of neurons and its deficiency leads to loss of primary cilia (2). **LGI1** (*leucine rich glioma inactivated 1*) functions as a negative regulator of excitatory synaptic transmission during post natal development in mice (3). Mice lacking **LGI1** display early-onset spontaneous seizures and increase in presynaptic glutamate release (3). It has been reported that assembly of primary cilia in dentate granule cells coincide with formation of glutamatergic synapses from these cells to entorhinal projections which mediate communication between hippocampus and neocortex (4). Dentate granule cells arise from adult hippocampal neurons located in the medial temporal lobe, a region in the brain from which epilepsy may originate, and integrate into existing neural circuits forming functional synapses (4). The novel interaction of **PKD2L1** with **LGI1** may throw light on the role played by cilia in etiology of epilepsy by influencing excitability of neurons.

Asymmetry is observed in structural aspects of the brain such as its cytoarchitecture, gray matter volume and integrity of white matter and in functional aspects of brain, viz. lateralization of cognitive processes such as the dominance of left hemisphere in language and right-handedness (5). This asymmetry which is critical in the functioning of a healthy brain is often reduced in Schizophrenia (6). In Schizophrenia, abnormal asymmetry in functional connections of the brain and disruption of left-hemisphere dominance are observed (6). Abnormalities in brain asymmetry have also been associated with bipolar disorder (7). The ciliary protein, **PKD1L1** (*polycystic kidney disease protein 1-like 1*) which forms a complex with PKD2 (*polycystic kidney disease 2 protein*) in motile cilia of Kupffer’s vesicle, has been shown to be involved in establishing left-right patterning (8). In zebrafish embryos, Kupffer’s vesicle has been previously identified as a ciliated organ which initiates left-right patterning in brain, gut and heart (9). The novel interactor **ABCA13** has been predicted to interact with **PKD1L1** (figure). Cytogenetic disruption, non-synonymous mutations and SNPs from GWAS data have indicated that **ABCA13** (*ATP binding cassette subfamily A member 13*) may be involved in Schizophrenia and bipolar disorder (10, 11). Even though the functions of **ABCA13** are yet to be elucidated, it has been found to be highly expressed in choroid plexus and ciliated ependymal cells lining the ventricles of the brain (10). The novel interaction of **PKD1L1** with **ABCA13** may point at processes underlying brain asymmetry during early stages of development, which may be disrupted in Schizophrenia and bipolar disorder due dysfunction of motile cilia.

Parkinson’s disease is associated with loss of dopaminergic neurons in a region of the brain called substantia nigra leading to loss of dopaminergic input to the striatum (12). A region in the brain of the model organism *Oryzias latipes* (commonly referred to as medaka) called the ventral telencephalon, which is innervated by dopaminergic neurons arising from diencephalon, has been equated in function to the human striatum (13). It has been reported that in medaka, a loss of the gene **PARK2** (*Parkinson protein 2 E3 ubiquitin protein ligase* or *Parkin*) leads to a loss of dopaminergic neurons in the middle diencephalon accompanied by a loss of dopaminergic fibres in the telencephalon (14). Overexpression of PARK2 (Log2FC=1.78, p-value=3.10E-16) was noted in 16 weeks old (fetal stage) diencephalons from right side of female embryos versus Carnegie stage 23 (the last embryonic stage) (15). Mutations in **PARK2** are associated with Parkinson’s disease (16). Loss of **PARK2** activity has been shown to decrease the number of dopaminergic neurons in posterior tuberculum of *Danio rerio* (commonly called zebrafish), a region that has been equated with substantia nigra in humans (13). This decrease in the number of dopaminergic neurons was enhanced on treatment with MPP+, a metabolite of MPTP (*1-methyl-4-phenyl-1,2,3,6-tetrahydropyridine*), which destroys dopaminergic neurons in substantia nigra and is commonly used to induce Parkinson’s disease in animal models (13). It has also been shown that motor deficits and loss of dopaminergic neurons induced by MPTP in mice are rescued by *Parkin*, delivered into the substantia nigra of mice using viral vectors (17). Interestingly, hemi-Parkinsonian rats having unilateral lesions in the dopamine pathway extending from substantia nigra to the striatum were found to have significantly longer neuronal cilia on the lesioned side than on the non-lesioned side (18). Mice injected with Reserpine, a drug that reduces dopamine levels, were found to have elongated neuronal cilia in the striatum and treatment with DRD2 (*dopamine receptor D2* ) agonists reverses this effect (18). Mice lacking DRD2 also exhibited longer neuronal cilia (18). Hence, the novel interaction of the ciliary protein **DYNLT1** with **PARK2** may point at mechanisms controlling the length of cilia on striatal neurons in Parkinson’s disease in which the dopaminergic input from substantia nigra to striatum is disrupted, possibly influenced by the loss of **PARK2** (figure). The cilia gene, **DYNLT1** (*dynein light chain Tctex-type 1*) also known as **Tctex-1**, has been identified as a component of the dynein-2 complex involved in regulating elongation of primary cilia (19). Ciliary length was found to increase when **Tctex-1** was lost following the suppression of dynein heavy chain-2 in retinal pigmented cells, which are used to model normal cilia on neural cells and ciliary defects in ciliopathies (19). Overexpression of DYNLT1 (Log2FC=0.29, p-value=0.0069) was noted in diencephalons from left side of male embryos at Carnegie stage 23 versus Carnegie stage 22 (15).

The novel interaction of **Tctex-1** with **PARK2** may also be relevant in another aspect of Parkinson’s disease etiology, viz. accumulation of the protein alpha-synuclein in lewy bodies. Lewy bodies are inclusion bodies rich in misfolded proteins, found in different regions of the brain of Parkinson’s disease patients (20). Alpha-synuclein accumulates in midbrain dopamine neurons lacking *Parkin*, derived from patient iPSCs (induced pluripotent stem cells) (21). Accumulation of alpha-synuclein has been noted in sub-ependymal and ependymal regions in the brains of Parkinson’s disease patients (22). Ependymal cells are ciliated and line cavities in the brain called ventricles, through which the cerebrospinal fluid flows (23). Ciliary defects in ependymal cells have been linked to development of hydrocephalus (23). It is interesting to note that increased levels of endogenous **Tctex-1** activity and **Tctex-1** promoter or enhancer activity are found in ependymal cells lining the third ventricle (24). It has also been reported that the gene PACRG (*parkin co-regulated gene*) which shares a bidirectional promoter with **PARK2** and also accumulates in lewy bodies of Parkinson’s disease patients, localizes to motile cilia lining the ventricles of the brain, specifically to ciliary axoneme (25). The study demonstrated that in mice, loss of PACRG results in impaired ciliary beating, impaired flow of cerebrospinal fluid mediated by cilia and development of hydrocephalus (25). Hence, the novel interaction of **Tctex-1** with **PARK2**, in this case, may point at processes that influence accumulation of alpha-synuclein in regions of the brain such as the ependyma that has motile cilia, viz. ciliary defects contributing to pathological manifestations of Parkinson’s disease.

## Novel PPIs involved in ciliopathy-associated hydrocephalus

Hydrocephalus is a condition frequently associated with ciliopathies in which cerebrospinal fluid accumulate in the brain ventricle giving rise to an enlarged head (26). While it has been established that defects in adhesion of ependymal cells and formation of cilia may lead to hydrocephalus, the exact mechanisms underlying it are yet to be elucidated (23). The novel interactor **IFITM1** interacting with two ciliary proteins, **NME5** and **DYNLT3**, other novel interactors of NME5, viz. **HBEGF**, **SPOCK1**, **MKRN1**, **APTX**, **VIPR1**, **VDAC1**, **P4HA2**, and the novel interactor **UIMC1** interacting with **DYNLT3** may open up the possibility of multiple factors colluding to cause hydrocephalus- broadly, the dysfunction of cilia in ependymal cells of the brain and a de-regulation of angiogenic factors that influence the formation of vascular lumen (figure). In this respect, loss of cilia among other markers of ependymal cells is observed when VEGF (*vascular endothelial growth factor*)- an inducer of angiogenesis- is infused into the ventricles of the brain, resulting in ventriculomegaly which mimics acquired hydrocephalus (27).

Interestingly, **NME5**, **UIMC1** and **DYNLT3** were predicted as novel interactors for **IFITM1** in our interactome of IFITMs (*interferon-induced transmembrane proteins*) which have been shown to inhibit infection of Zika virus (28). Based on functions of the interacting proteins, a testable hypothesis which linked cilia dysfunction in ependymal cells to microcephaly- a fetal malformation associated with Zika virus infection- was presented in this study (28). Microcephaly is the condition in which the head circumference of newborn babies is below the second percentile for gestation (29). It has previously been suggested that genes implicated in microcephaly may also influence the development of hydrocephalus. For example, ASPM (*abnormal spindle-like microcephaly-associated protein*) which is associated with primary microcephaly and has undergone rapid evolution over the course of recent evolution of hominids is implicated in the size of cerebral cortex and neurogenesis (30). ASH domains that are normally found in proteins localizing to cilia such as *Hydin* and OCRL were also identified in ASPM and this has indicated that ASPM may be involved in cilia dysfunction and hydrocephalus (31). *Hydin* and OCRL have been associated with microcephaly and Lowe oculocerebrorenal syndrome respectively (32, 33). Hence, further investigations into shared mechanisms between development of hydrocephalus and microcephaly in the context of cilia dysfunction are necessary.

**NME5** (*non-metastatic cells 5*) is highly expressed in ependymal cells. In mice homozygous for **NME5**, moderate to marked hydrocephalus along with ciliary dysfunction has been observed (34). **NME5** has been reported to localize within the axoneme in a manner dependent on RIBC2 (*RIB43A domain with coiled-coils 2*), which has been implicated in ciliary motion (35). RIBC2 was predicted here as a novel interactor for ATXN10 (*ataxin 10*), mutations in which were associated with the ciliopathy, nephronophthisis (36). Cytoskeletal events that are guided by VEGF influence formation of vascular lumens (37). It was reported that excess of **HBEGF**- a novel interactor of **NME5**- leads to significant elevation of VEGF and dilation of ventricles, leading to hydrocephalus (38). **HBEGF** (*heparin binding EGF like growth factor*) is known to localize to the ventricular zone and the cortical layer during embryonic development and mediates radial migration in the developing brain (39). HBEGF was found to be overexpressed (Log2FC=2.22, p-value=1.50E-08) in 11 weeks old choroid plexuses of female embryos versus Carnegie stage 23.(15) The choroid plexus, secreting cerebrospinal fluid in the brain, consists of modified, ciliated ependymal cells. **HBEGF** has also been reported to activate eNOS (*endothelial nitric oxide synthase*) which plays a critical role in angiogenesis induced by VEGF and vascular hyperpermeability and is a known interactor of DYNLLI (*dynein light chain LC8-type 1*) in the cilia interactome (40, 41). **IFITM1**, another novel interactor of **NME5**, is expressed in endothelial cells of the bladder, brain and stomach in a manner which is correlated with maturation of blood vessels (42). While being induced during maturation stages of angiogenesis in vitro, in vivo **IFITM1** is stably expressed by microvascular endothelial cells which are quiescent (42). IFITM1 was found to be overexpressed (Log2FC=2.52, p-value=1.10E-09) in 13 weeks old choroid plexuses of female embryos versus Carnegie stage 23 (15). The fact that **IFITM1** (*interferon induced transmembrane protein 1*) was involved in establishing stable contacts between cells during formation of lumen in endothelial tissue was revealed by a study. On knockdown of **IFITM1**, OCLN (occludin) which normally interacts with **IFITM1** and localizes to tight junctions between endothelial cells, is mislocalized as a result of which intercellular vacuoles fail to fuse and form multicellular lumen (42). The mechanism by which **IFITM1** regulates assembly of tight junctions was speculated to be related to endosomal trafficking as it is from recycling endosomes that internalized OCLN is returned to the plasma membrane during remodeling of tight junctions in endothelial cells. **IFITM1** was also predicted as a novel interactor of **DYNLT3** (*dynein light chain Tctex-type 3*) which has been suggested to be associated with dynein 2 (43). *Dynein 2*, which has been associated with human skeletal ciliopathies, is known to be involved in intraflagellar transport (44). Moreover, high expression of *dynein 2* has been observed in ependymal cells (45). Another novel interactor predicted for **DYNLT3** is **UIMC1**. Depletion of **UIMC1** (*ubiquitin interaction motif containing 1*) which occurs in a complex with p73 when overexpressed, impairs translocation of BRCA1 (*breast cancer 1*) to DNA damage sites resulting in defective control of cell cycle and repair of double strand breaks (46, 47). p73 has been implicated in the development and maintenance of ependymal cells and in animals deficient in p73, increased apoptosis and lack of differentiation of RGCs into ependymal cells accompanied by loss of motile cilia resulting in hydrocephalus and hippocampal dysgenesis were reported (48, 49). In a recent study it was observed that p73 is required for establishing translational polarity of the basal body patches on the apical surface of ependymal cells and that disruption of p73 resulting in abnormal translational polarity models human congenital hydrocephalus (50).

Loss of **APTX**, a novel interactor of **NME5**, which is involved in the repair of single stranded DNA, accelerates senescence and delays transcription recovery in cultured cells (51). **APTX** (*aprataxin*) is known to interact with CEP350 (*centrosomal protein 350*) which localizes to the basal body in ciliated cells and is required for ciliogenesis (52). **APTX** also interacts with CNTROB (*centrobin*), a centrosomal protein that is required for the elongation and stability of centrioles (53). Centrosomes assist in assembling primary cilia in quiescent cells (54). Downregulation of the centrosomal protein **VDAC1** (*voltage dependent anion channel 1*) that localizes to the mother centriole gives rise to abnormal ciliogenesis while overexpression of **VDAC1** suppresses cilia formation (55). Low levels of VEGF-A, destructuration of blood vessels and inflammation observed in *Vdac1−/−* RAS MEF tumors also implicate **VDAC1** in vascular development (56). **VDAC1** interacts with several MMPs (*matrix metalloproteinases*) that influence angiogenesis induced by VEGF such as MMP2, MMP3 and MMP14. The expression of MMP2 was significantly higher along with VEGF-A in endothelial cells affected with arteriovenous malformations in the human brain (57). The expression of MMP3 was associated with expression of VEGF-A and its circulating levels in healthy adults (58). The enzymatic activity of MMP14 which interacts with VEGFR1 was found to be necessary for angiogenesis induced by VEFG-A (59). Another novel interactor of **NME5**, **SPOCK1** (*SPARC/osteonectin, Cwcv And Kazal like domains proteoglycan 1*), associated with developmental delay and microcephaly, is strongly expressed in the brain where it modulates neurogenesis and axonal growth during early stages of development (60, 61). SPOCK1 was found to be overexpressed (Log2FC=1.82, p-value=4.60E-10) in 13 weeks old choroid plexuses of female embryos versus Carnegie stage 23 (15). **VIPR1** (*vasoactive intestinal peptide receptor 1*), a novel interactor of **NME5**, has been reported to increase expression of VEGF promoting proliferation of endothelial cells in the vascular tissue of brain via the CAMP/PKA pathway after ischemic insult in vitro (62). **P4HA2** (*prolyl 4-hydroxylase subunit alpha 2*), a novel interactor of **NME5**, is a target gene of the angiogenic process and a hypoxia response gene which was described as the genetic and biochemical link between the p53 tumor suppressor pathway and the synthesis of collagen fragments such as endostatin and tumstatin which are anti-angiogenic (63, 64). The subventricular zone (SVZ) is separated from the cerebrospinal fluid (CSF) flowing inside the lateral ventricles by the layer of ependymal cells that have cilia (65). Both change in the SVZ niche and the integrity of this ependymal boundary are correlated with age (65). Therefore the expression of TERT (*telomerase reverse transcriptase*) that is required for maintaining proliferative capacity of cells is reduced in an age-dependent manner in the SVZ with decreased expression of TERT correlating with decreased neurogenesis in SVZ (66-68). **MKRN1**, a novel interactor of **NME5**, is known to modulate the length of telomeres through proteolysis of TERT which is a known interactor of a ciliary protein DYNLL1 implicated in cell survival, in the cilia interactome (69, 70). **MKRN1** (*Makorin ring finger protein 1*) is also known to interact with MAP1LC3B which is a microtubule-associated protein that localizes to the ciliary axoneme (71).

## Novel PPI involved in primary ciliary dyskinesia

It is known that motile cilia in the respiratory tract participate in mucociliary clearance, i.e. the transport of mucus with trapped microbes and harmful particles away from lungs to protect them from infections and injury (72). In Primary Ciliary Dyskinesia (PCD), a ciliopathy arising from dysfunction of motile cilia and characterized by respiratory tract infections, situs defects and infertility, mucociliary clearance is defective due to cilia dysfunction (73). This leads to recurrent infections in the respiratory tracts of PCD patients (73). Elevated levels of inflammatory cytokines including **IL6** (*interleukin 6*), associated with bacterial infections, have been observed in the nasal cavity of PCD patients (74). **IL6**, which is usually secreted in response to infectious agents or toxic particles, is upregulated in respiratory diseases such as asthma and chronic obstructive pulmonary disease (75). In our interactome, **IL6** has been predicted as a novel interactor of **DNAH11**, which is implicated in PCD (figure). **DNAH11** (*dynein axonemal heavy chain 11*) localizes to the respiratory cilia and mutations in **DNAH11** disrupt the utrastructure of normal cilia and cause hyperkinetic ciliary beating in PCD (76). **IL6** has been identified as a positive regulator of multiciliogenesis during airway repair (77). **IL6** secreted by mesenchymal stromal cells after airway injury activates the STAT3 (*signal transducer and activator of transcription 3*) pathway, promoting regeneration of cells and ciliogenesis by inhibiting the Notch pathway and regulating genes involved in ciliogenesis such as FOXJ1 (77). **IL6** also appears to have an inhibitory effect on beating frequency of cilia in fallopian tubes (78). The novel interaction of **DNAH11** with **IL6** may provide clues to the etiology of respiratory tract defects in PCD, viz. the contribution of inflammatory processes to cilia dysfunction.

## Novel PPIs involved in ciliogenesis

Ciliogenesis and progression of cell cycle are inversely related (79-81). Ciliogenesis has been shown to commence when cells enter their quiescence phase known as G0 phase. On the other hand, cilia are resorbed at the G0/G1 transition phase before cells re-enter the cell cycle and start dividing. The novel interactions of the ciliary protein **CDK3** with **FOXJ1**, **TLK2** and **NAP1L1** may throw light on the cross talk between ciliogenesis and the cell cycle (figure). **CDK3** (*cyclin dependent kinase 3*) promotes exit of cells from their quiescent phase and re-entry into the cell cycle through pRB (*retinoblastoma protein*) phosphorylation (82). **FOXJ1** (*forkhead box J1*), a novel interactor of **CDK3**, is a transcription factor that activates genes involved in ciliogenesis and its activity has been shown to be sufficient for ectopic formation of motile cilia in Xenopus and Zebrafish (83). **TLK2** (*tousled like kinase 2*) regulates the S phase or synthesis phase of the cell cycle by phosphorylating the histone chaperones, ASF1A (*anti-silencing function 1A histone chaperone*) and ASF1B (*anti-silencing function 1B histone chaperone*), which insert histone dimers into newly synthesized DNA (84). **NAP1L1** (*nucleosome assembly protein 1 like 1*) has been reported to be essential for centriole duplication induced by PLK4 (*polo like kinase 4*), a master regulator of centriole formation whose overexpression leads to loss of primary cilia and an increase in the number of proliferating cells (85, 86). It is known that centrioles play an important role in ciliogenesis by acting as basal bodies that give rise to cilia (87).

In early phases of ciliogenesis, actin cytoskeleton has to be re-modeled to form a dense network of actin molecules or an “actin net” at the apical membrane, to allow docking of basal bodies and formation of axonemes (88). It has been shown that RHOA (*ras homolog family member A*) plays an important role in establishing this actin net in ciliated cells (88). **CDK18**, a novel interactor of the ciliary protein **DNAH2**, has been shown to negatively regulate actin cytoskeleton via the FAK/Rho signaling pathway (89). Knockdown of **CDK18** (*cyclin dependent kinase 18*) led to accumulation of actin at the leading edge of HEK293T cells which showed enhanced cell motility and RhoA/Rho-associated kinase activity, while overexpression of **CDK18** led to the formation of filopodia (cellular projections rich in F-actin) in HeLa cells (89, 90). **CDK18** was reported to be upregulated during development of cardiomyocytes from proliferative phases to a stage in which the growth of cardiomyocytes was arrested (91). **CDK18** was also found to be significantly upregulated (log2 FC= 2.0027) in embryoid bodies with beating cardiomyocytes in comparison with embryonic stem cells (92). **CDK18** was found to interact with Cyclin K, which has been found in a complex with CDK13, implicated in syndromic congenital heart disease (93-95). In our interactome, **CDK18** has been predicted to interact with **DNAH2** (*dynein axonemal heavy chain 2*) , a ciliary protein localized to inner arms of the cilium axoneme (96). Hence, the novel interaction of **DNAH2** with **CDK18** may point at processes underlying ciliogenesis during heart development and may also help in unraveling functions of **CDK18** which belongs to the family of highly conserved cyclin-dependent kinases called PCTAIRE kinases that are poorly understood (figure) (97). Since development of heart and brain progresses in tandem sharing common morphogenetic programs, the novel interaction of **DNAH2** with **CDK18** may also point at processes orchestrating ciliogenesis in the brain (98). Interestingly, it was noted from the Human Protein Atlas that **CDK18** had elevated expression in both heart muscle (65.7 transcripts per million) and cerebral cortex (60.7 transcripts per million) (99).

The novel interaction of **ARL6** with the ciliary protein **ARL13B** may be involved in regulating cilia length during ciliogenesis (figure). While overexpression of the ciliary protein implicated in ciliogenesis **ARL13B** (*ADP ribosylation factor like GTPase 13B*) increases cilia length, its loss has been shown to reduce cilia length (100). **ARL6** (*ADP ribosylation factor like GTPase 6*) is a GTP-binding protein mutated in Bardet-Biedl syndrome (101). Proteins that regulate ciliary length have also been shown to be responsible for maintaining protein trafficking in cilia under steady state conditions (102). In this respect, **ARL6** is involved in trafficking proteins of the Bardet-Biedl syndrome complex and also in regulating cilia length (103).

## Novel PPI involved in trafficking of membrane receptors in cilia

BBS proteins, which are implicated in Bardet-Biedl syndrome, have been found to be required for localizing GPCRs to primary cilia on neurons (104). GPCRs (*G protein-coupled receptors*) are membrane receptors that are modulated by >30% of all the drugs that are commercially available (105). The GPCR, **QRFPR** (*pyroglutamylated RFamide peptide receptor*), is a ciliary protein which has been predicted to interact with the novel interactor **BBS12** (figure). **QRFPR** localizes to primary cilia on embryonic hypothalamic neurons in rats and also to cilia on retinal pigmented cells (106). **BBS12** (*Bardet-Biedl syndrome 12*), the novel interactor of **QRFPR**, forms a complex with BBS6 and BBS10 which mediates BBSome assembly (107). **BBS12** also interacts with BBS4 (107). The GPCR, rhodopsin, has been found to accumulate in the cell bodies of photoreceptors in mice lacking BBS4 and BBS6.(108, 109) Two GPCRS, viz. SSTR3 (*somatostatin receptor 3*) and MCHR1 (*melanin concentrating hormone receptor 1*), fail to localize to neuronal cilia in mice lacking BBS4 (104). The novel interaction of **QRFPR** with **BBS12** may open up the possibility of **BBS12** mediating localization of the GPCR, **QRFPR**, into neuronal cilia.

References

1. Gu, W., Brodtkorb, E., and Steinlein, O. K. (2002) LGI1 is mutated in familial temporal lobe epilepsy characterized by aphasic seizures. *Annals of neurology* 52, 364-367

2. Yao, G., Luo, C., Harvey, M., Wu, M., Schreiber, T. H., Du, Y., Basora, N., Su, X., Contreras, D., and Zhou, J. (2015) Disruption of polycystin-L causes hippocampal and thalamocortical hyperexcitability. *Human molecular genetics* 25, 448-458

3. Boillot, M., Lee, C.-Y., Allene, C., Leguern, E., Baulac, S., and Rouach, N. (2016) LGI1 acts presynaptically to regulate excitatory synaptic transmission during early postnatal development. *Scientific reports* 6, 21769

4. Kumamoto, N., Gu, Y., Wang, J., Janoschka, S., Takemaru, K.-I., Levine, J., and Ge, S. (2012) A role for primary cilia in glutamatergic synaptic integration of adult-born neurons. *Nature neuroscience* 15, 399-405

5. Rentería, M. E. (2012) Cerebral asymmetry: a quantitative, multifactorial, and plastic brain phenotype. *Twin Research and Human Genetics* 15, 401-413

6. Ribolsi, M., Daskalakis, Z. J., Siracusano, A., and Koch, G. (2014) Abnormal asymmetry of brain connectivity in schizophrenia. *Frontiers in human neuroscience* 8

7. Reite, M., Teale, P., Rojas, D. C., Arciniegas, D., and Sheeder, J. (1999) Bipolar disorder: anomalous brain asymmetry associated with psychosis. *American Journal of Psychiatry* 156, 1159-1163

8. Kamura, K., Kobayashi, D., Uehara, Y., Koshida, S., Iijima, N., Kudo, A., Yokoyama, T., and Takeda, H. (2011) Pkd1l1 complexes with Pkd2 on motile cilia and functions to establish the left-right axis. *Development* 138, 1121-1129

9. Essner, J. J., Amack, J. D., Nyholm, M. K., Harris, E. B., and Yost, H. J. (2005) Kupffer9s vesicle is a ciliated organ of asymmetry in the zebrafish embryo that initiates left-right development of the brain, heart and gut. *Development* 132, 1247-1260

10. Knight, H. M., Pickard, B. S., Maclean, A., Malloy, M. P., Soares, D. C., McRae, A. F., Condie, A., White, A., Hawkins, W., and McGhee, K. (2009) A cytogenetic abnormality and rare coding variants identify ABCA13 as a candidate gene in schizophrenia, bipolar disorder, and depression. *The American Journal of Human Genetics* 85, 833-846

11. O'donovan, M. C., Craddock, N., Norton, N., Williams, H., Peirce, T., Moskvina, V., Nikolov, I., Hamshere, M., Carroll, L., and Georgieva, L. (2008) Identification of loci associated with schizophrenia by genome-wide association and follow-up. *Nature genetics* 40, 1053

12. Huot, P., Lévesque, M., and Parent, A. (2006) The fate of striatal dopaminergic neurons in Parkinson's disease and Huntington's chorea. *Brain* 130, 222-232

13. Flinn, L., Mortiboys, H., Volkmann, K., Köster, R. W., Ingham, P. W., and Bandmann, O. (2009) Complex I deficiency and dopaminergic neuronal cell loss in parkin-deficient zebrafish (Danio rerio). *Brain* 132, 1613-1623

14. Matsui, H., Gavinio, R., Asano, T., Uemura, N., Ito, H., Taniguchi, Y., Kobayashi, Y., Maki, T., Shen, J., and Takeda, S. (2013) PINK1 and Parkin complementarily protect dopaminergic neurons in vertebrates. *Human molecular genetics* 22, 2423-2434

15. Lindsay, S. J., Xu, Y., Lisgo, S. N., Harkin, L. F., Copp, A. J., Gerrelli, D., Clowry, G. J., Talbot, A., Keogh, M. J., and Coxhead, J. (2016) HDBR expression: a unique resource for global and individual gene expression studies during early human brain development. *Frontiers in neuroanatomy* 10, 86

16. Nuytemans, K., Theuns, J., Cruts, M., and Van Broeckhoven, C. (2010) Genetic etiology of Parkinson disease associated with mutations in the SNCA, PARK2, PINK1, PARK7, and LRRK2 genes: a mutation update. *Human mutation* 31, 763-780

17. Yasuda, T., Hayakawa, H., Nihira, T., Ren, Y.-R., Nakata, Y., Nagai, M., Hattori, N., Miyake, K., Takada, M., and Shimada, T. (2011) Parkin-mediated protection of dopaminergic neurons in a chronic MPTP-minipump mouse model of Parkinson disease. *Journal of Neuropathology & Experimental Neurology* 70, 686-697

18. Miyoshi, K., Kasahara, K., Murakami, S., Takeshima, M., Kumamoto, N., Sato, A., Miyazaki, I., Matsuzaki, S., Sasaoka, T., and Katayama, T. (2014) Lack of dopaminergic inputs elongates the primary cilia of striatal neurons. *PloS one* 9, e97918

19. Palmer, K. J., MacCarthy-Morrogh, L., Smyllie, N., and Stephens, D. J. (2011) A role for Tctex-1 (DYNLT1) in controlling primary cilium length. *European journal of cell biology* 90, 865-871

20. Kim, W. S., Kågedal, K., and Halliday, G. M. (2014) Alpha-synuclein biology in Lewy body diseases. *Alzheimer's research & therapy* 6, 73

21. Chung, S. Y., Kishinevsky, S., Mazzulli, J. R., Graziotto, J., Mrejeru, A., Mosharov, E. V., Puspita, L., Valiulahi, P., Sulzer, D., and Milner, T. A. (2016) Parkin and PINK1 patient iPSC-derived midbrain dopamine neurons exhibit mitochondrial dysfunction and α-synuclein accumulation. *Stem cell reports* 7, 664-677

22. Kovacs, G. (2014) Clinical Neuropathology image 5-2014: α-synuclein pathology in the ependyma in Parkinson’s disease. *Clinical neuropathology* 33, 328

23. Jiménez, A. J., Domínguez-Pinos, M.-D., Guerra, M. M., Fernández-Llebrez, P., and Pérez-Fígares, J.-M. (2014) Structure and function of the ependymal barrier and diseases associated with ependyma disruption. *Tissue barriers* 2, e28426

24. Sung, C.-H., and Chuang, J.-Z. (2014) Tctex-1 regulatory sequence as stem cell marker. Google Patents

25. Wilson, G. R., Wang, H. X., Egan, G. F., Robinson, P. J., Delatycki, M. B., O'bryan, M. K., and Lockhart, P. J. (2010) Deletion of the Parkin co-regulated gene causes defects in ependymal ciliary motility and hydrocephalus in the quaking viable mutant mouse. *Human molecular genetics* 19, 1593-1602

26. Fliegauf, M., Benzing, T., and Omran, H. (2007) When cilia go bad: cilia defects and ciliopathies. *Nature reviews Molecular cell biology* 8, 880-893

27. Shim, J. W., Sandlund, J., and Madsen, J. R. (2014) VEGF: a potential target for hydrocephalus. *Cell and tissue research* 358, 667-683

28. Ganapathiraju, M. K., Karunakaran, K. B., and Correa-Menéndez, J. (2017) Predicted protein interactions of IFITMs may shed light on mechanisms of Zika virus-induced microcephaly and host invasion. *F1000Research* 5

29. Mlakar, J., Korva, M., Tul, N., Popović, M., Poljšak-Prijatelj, M., Mraz, J., Kolenc, M., Resman Rus, K., Vesnaver Vipotnik, T., and Fabjan Vodušek, V. (2016) Zika virus associated with microcephaly. *N Engl J Med* 2016, 951-958

30. Kouprina, N., Pavlicek, A., Mochida, G. H., Solomon, G., Gersch, W., Yoon, Y.-H., Collura, R., Ruvolo, M., Barrett, J. C., and Woods, C. G. (2004) Accelerated evolution of the ASPM gene controlling brain size begins prior to human brain expansion. *PLoS biology* 2, e126

31. Ponting, C. P. (2006) A novel domain suggests a ciliary function for ASPM, a brain size determining gene. *Bioinformatics* 22, 1031-1035

32. Swan, L. E., Tomasini, L., Pirruccello, M., Lunardi, J., and De Camilli, P. (2010) Two closely related endocytic proteins that share a common OCRL-binding motif with APPL1. *Proceedings of the National Academy of Sciences* 107, 3511-3516

33. Dawe, H. R., Shaw, M. K., Farr, H., and Gull, K. (2007) The hydrocephalus inducing gene product, Hydin, positions axonemal central pair microtubules. *BMC biology* 5, 33

34. Vogel, P., Read, R., Hansen, G., Payne, B., Small, D., Sands, A., and Zambrowicz, B. (2012) Congenital hydrocephalus in genetically engineered mice. *Veterinary pathology* 49, 166-181

35. Chung, M.-I., Kwon, T., Tu, F., Brooks, E. R., Gupta, R., Meyer, M., Baker, J. C., Marcotte, E. M., and Wallingford, J. B. (2014) Coordinated genomic control of ciliogenesis and cell movement by RFX2. *Elife* 3, e01439

36. Sang, L., Miller, J. J., Corbit, K. C., Giles, R. H., Brauer, M. J., Otto, E. A., Baye, L. M., Wen, X., Scales, S. J., and Kwong, M. (2011) Mapping the NPHP-JBTS-MKS protein network reveals ciliopathy disease genes and pathways. *Cell* 145, 513-528

37. Lammert, E., and Axnick, J. (2012) Vascular lumen formation. *Cold Spring Harbor perspectives in medicine* 2, a006619

38. Shim, J. W., Sandlund, J., Hameed, M. Q., Blazer-Yost, B., Zhou, F. C., Klagsbrun, M., and Madsen, J. R. (2016) Excess HB-EGF, which promotes VEGF signaling, leads to hydrocephalus. *Scientific reports* 6, 26794

39. Caric, D., Raphael, H., Viti, J., Feathers, A., Wancio, D., and Lillien, L. (2001) EGFRs mediate chemotactic migration in the developing telencephalon. *Development* 128, 4203-4216

40. Mehta, V. B., Zhou, Y., Radulescu, A., and Besner, G. E. (2008) HB-EGF stimulates eNOS expression and nitric oxide production and promotes eNOS dependent angiogenesis. *Growth factors* 26, 301-315

41. Fukumura, D., Gohongi, T., Kadambi, A., Izumi, Y., Ang, J., Yun, C.-O., Buerk, D. G., Huang, P. L., and Jain, R. K. (2001) Predominant role of endothelial nitric oxide synthase in vascular endothelial growth factor-induced angiogenesis and vascular permeability. *Proceedings of the National Academy of Sciences* 98, 2604-2609

42. Popson, S. A., Ziegler, M. E., Chen, X., Holderfield, M. T., Shaaban, C. I., Fong, A. H., Welch-Reardon, K. M., Papkoff, J., and Hughes, C. C. (2014) Interferon-induced transmembrane protein 1 regulates endothelial lumen formation during angiogenesis. *Arteriosclerosis, thrombosis, and vascular biology*, ATVBAHA. 114.303352

43. Hou, Y., and Witman, G. B. (2015) Dynein and intraflagellar transport. *Experimental cell research* 334, 26

44. Schmidt, H., Zalyte, R., Urnavicius, L., and Carter, A. P. (2015) Structure of human cytoplasmic dynein-2 primed for its powerstroke. *Nature* 518, 435

45. Mikami, A., Tynan, S. H., Hama, T., Luby-Phelps, K., Saito, T., Crandall, J. E., Besharse, J. C., and Vallee, R. B. (2002) Molecular structure of cytoplasmic dynein 2 and its distribution in neuronal and ciliated cells. *Journal of cell science* 115, 4801-4808

46. Yan, J., and Jetten, A. M. (2008) RAP80 and RNF8, key players in the recruitment of repair proteins to DNA damage sites. *Cancer letters* 271, 179-190

47. Marshall, C. B. (2016) The Essential Role of p73 in Multiciliated Cell Development.

48. Gonzalez‐Cano, L., Fuertes‐Alvarez, S., Robledinos‐Anton, N., Bizy, A., Villena‐Cortes, A., Farinas, I., Marques, M., and Marin, M. C. (2016) p73 is required for ependymal cell maturation and neurogenic SVZ cytoarchitecture. *Developmental neurobiology* 76, 730-747

49. Marshall, C. B., Mays, D. J., Beeler, J. S., Rosenbluth, J. M., Boyd, K. L., Guasch, G. L. S., Shaver, T. M., Tang, L. J., Liu, Q., and Shyr, Y. (2016) p73 is required for multiciliogenesis and regulates the Foxj1-associated gene network. *Cell reports* 14, 2289-2300

50. Fujitani, M., Sato, R., and Yamashita, T. (2017) Loss of p73 in ependymal cells during the perinatal period leads to aqueductal stenosis. *Scientific Reports* 7, 12007

51. Carroll, J., Page, T. K., Chiang, S.-C., Kalmar, B., Bode, D., Greensmith, L., Mckinnon, P. J., Thorpe, J. R., Hafezparast, M., and El-Khamisy, S. F. (2014) Expression of a pathogenic mutation of SOD1 sensitizes aprataxin-deficient cells and mice to oxidative stress and triggers hallmarks of premature ageing. *Human molecular genetics* 24, 828-840

52. Mojarad, B. A., Gupta, G. D., Hasegan, M., Goudiam, O., Basto, R., Gingras, A.-C., and Pelletier, L. (2017) CEP19 cooperates with FOP and CEP350 to drive early steps in the ciliogenesis programme. *Open biology* 7, 170114

53. Gudi, R., Zou, C., Li, J., and Gao, Q. (2011) Centrobin–tubulin interaction is required for centriole elongation and stability. *The Journal of cell biology* 193, 711-725

54. Majumder, S., and Fisk, H. A. (2013) VDAC3 and Mps1 negatively regulate ciliogenesis. *Cell Cycle* 12, 849-858

55. Majumder, S., Cash, A., and Fisk, H. A. (2015) Non-overlapping distributions and functions of the VDAC family in ciliogenesis. *Cells* 4, 331-353

56. Brahimi-Horn, M. C., Giuliano, S., Saland, E., Lacas-Gervais, S., Sheiko, T., Pelletier, J., Bourget, I., Bost, F., Féral, C., and Boulter, E. (2015) Knockout of Vdac1 activates hypoxia-inducible factor through reactive oxygen species generation and induces tumor growth by promoting metabolic reprogramming and inflammation. *Cancer & metabolism* 3, 8

57. Xu, M., Xu, H., Qin, Z., Zhang, J., Yang, X., and Xu, F. (2014) Increased expression of angiogenic factors in cultured human brain arteriovenous malformation endothelial cells. *Cell biochemistry and biophysics* 70, 443-447

58. Saleh, A., Stathopoulou, M. G., Dadé, S., Ndiaye, N. C., Azimi-Nezhad, M., Murray, H., Masson, C., Lamont, J., Fitzgerald, P., and Visvikis-Siest, S. (2015) Angiogenesis related genes NOS3, CD14, MMP3 and IL4R are associated to VEGF gene expression and circulating levels in healthy adults. *BMC medical genetics* 16, 90

59. Han, K.-Y., Dugas-Ford, J., Lee, H., Chang, J.-H., and Azar, D. T. (2015) MMP14 Cleavage of VEGFR1 in the Cornea Leads to a VEGF-Trap Antiangiogenic EffectMMP14 Processing of VEGFR1. *Investigative ophthalmology & visual science* 56, 5450-5456

60. Röll, S., Seul, J., Paulsson, M., and Hartmann, U. (2006) Testican-1 is dispensable for mouse development. *Matrix biology* 25, 373-381

61. Dhamija, R., Graham, J. M., Smaoui, N., Thorland, E., and Kirmani, S. (2014) Novel de novo SPOCK1 mutation in a proband with developmental delay, microcephaly and agenesis of corpus callosum. *European journal of medical genetics* 57, 181-184

62. Yang, J., Shi, Q.-D., Song, T.-B., Feng, G.-F., Zang, W.-J., Zong, C.-H., and Chang, L. (2013) Vasoactive intestinal peptide increases VEGF expression to promote proliferation of brain vascular endothelial cells via the cAMP/PKA pathway after ischemic insult in vitro. *Peptides* 42, 105-111

63. Berger, B., Capper, D., Lemke, D., Pfenning, P.-N., Platten, M., Weller, M., von Deimling, A., Wick, W., and Weiler, M. (2010) Defective p53 antiangiogenic signaling in glioblastoma. *Neuro-oncology* 12, 894-907

64. Gilkes, D. M., Bajpai, S., Chaturvedi, P., Wirtz, D., and Semenza, G. L. (2013) Hypoxia-inducible factor 1 (HIF-1) promotes extracellular matrix remodeling under hypoxic conditions by inducing P4HA1, P4HA2, and PLOD2 expression in fibroblasts. *Journal of Biological Chemistry* 288, 10819-10829

65. Conover, J. C., and Shook, B. A. (2011) Aging of the subventricular zone neural stem cell niche. *Aging and disease* 2, 49

66. Caporaso, G. L., Lim, D. A., Alvarez-Buylla, A., and Chao, M. V. (2003) Telomerase activity in the subventricular zone of adult mice. *Molecular and Cellular Neuroscience* 23, 693-702

67. Ferrón, S. R., Marqués-Torrejón, M. Á., Mira, H., Flores, I., Taylor, K., Blasco, M. A., and Farinas, I. (2009) Telomere shortening in neural stem cells disrupts neuronal differentiation and neuritogenesis. *Journal of Neuroscience* 29, 14394-14407

68. Ferrón, S., Mira, H., Franco, S., Cano-Jaimez, M., Bellmunt, E., Ramírez, C., Fariñas, I., and Blasco, M. A. (2004) Telomere shortening and chromosomal instability abrogates proliferation of adult but not embryonic neural stem cells. *Development* 131, 4059-4070

69. Kim, J. H., Park, S.-M., Kang, M. R., Oh, S.-Y., Lee, T. H., Muller, M. T., and Chung, I. K. (2005) Ubiquitin ligase MKRN1 modulates telomere length homeostasis through a proteolysis of hTERT. *Genes & development* 19, 776-781

70. Vadlamudi, R. K., Bagheri-Yarmand, R., Yang, Z., Balasenthil, S., Nguyen, D., Sahin, A. A., den Hollander, P., and Kumar, R. (2004) Dynein light chain 1, a p21-activated kinase 1-interacting substrate, promotes cancerous phenotypes. *Cancer cell* 5, 575-585

71. Lam, H. C., Cloonan, S. M., Bhashyam, A. R., Haspel, J. A., Singh, A., Sathirapongsasuti, J. F., Cervo, M., Yao, H., Chung, A. L., and Mizumura, K. (2013) Histone deacetylase 6–mediated selective autophagy regulates COPD-associated cilia dysfunction. *The Journal of clinical investigation* 123, 5212

72. Stannard, W., and O'Callaghan, C. (2006) Ciliary function and the role of cilia in clearance. *Journal of aerosol medicine* 19, 110-115

73. Tilley, A. E., Walters, M. S., Shaykhiev, R., and Crystal, R. G. (2015) Cilia dysfunction in lung disease. *Annual review of physiology* 77, 379-406

74. Shoemark, A., Frost, E., Harman, K., Crowley, S., Ives, A., Donovan, J., Tan, H.-L., Alton, E. W., Bush, A., and Hogg, C. (2017) Nasal cavity inflammation in patients with primary ciliary dyskinesia (PCD) is associated with bacterial infection. Eur Respiratory Soc

75. Rincon, M., and Irvin, C. G. (2012) Role of IL-6 in asthma and other inflammatory pulmonary diseases. *International journal of biological sciences* 8, 1281

76. Dougherty, G. W., Loges, N. T., Klinkenbusch, J. A., Olbrich, H., Pennekamp, P., Menchen, T., Raidt, J., Wallmeier, J., Werner, C., and Westermann, C. (2016) DNAH11 localization in the proximal region of respiratory cilia defines distinct outer dynein arm complexes. *American journal of respiratory cell and molecular biology* 55, 213-224

77. Tadokoro, T., Wang, Y., Barak, L. S., Bai, Y., Randell, S. H., and Hogan, B. L. (2014) IL-6/STAT3 promotes regeneration of airway ciliated cells from basal stem cells. *Proceedings of the National Academy of Sciences* 111, E3641-E3649

78. Papathanasiou, A., Djahanbakhch, O., Saridogan, E., and Lyons, R. (2008) The effect of interleukin-6 on ciliary beat frequency in the human fallopian tube. *Fertility and sterility* 90, 391-394

79. Keeling, J., Tsiokas, L., and Maskey, D. (2016) Cellular mechanisms of ciliary length control. *Cells* 5, 6

80. Plotnikova, O. V., Pugacheva, E. N., and Golemis, E. A. (2009) Primary cilia and the cell cycle. *Methods in cell biology* 94, 137-160

81. Goto, H., Inoko, A., and Inagaki, M. (2013) Cell cycle progression by the repression of primary cilia formation in proliferating cells. *Cellular and molecular life sciences* 70, 3893-3905

82. Ren, S., and Rollins, B. J. (2004) Cyclin C/cdk3 promotes Rb-dependent G0 exit. *Cell* 117, 239-251

83. Yu, X., Ng, C. P., Habacher, H., and Roy, S. (2008) Foxj1 transcription factors are master regulators of the motile ciliogenic program. *Nature genetics* 40, 1445-1453

84. Klimovskaia, I. M., Young, C., Strømme, C. B., Menard, P., Jasencakova, Z., Mejlvang, J., Ask, K., Ploug, M., Nielsen, M. L., and Jensen, O. N. (2014) Tousled-like kinases phosphorylate Asf1 to promote histone supply during DNA replication. *Nature communications* 5, 3394

85. Comartin, D. (2015) The role of CEP120 and SPICE1 in human centriole duplication. University of Toronto (Canada)

86. Coelho, P. A., Bury, L., Shahbazi, M. N., Liakath-Ali, K., Tate, P. H., Wormald, S., Hindley, C. J., Huch, M., Archer, J., and Skarnes, W. C. (2015) Over-expression of Plk4 induces centrosome amplification, loss of primary cilia and associated tissue hyperplasia in the mouse. *Open biology* 5, 150209

87. Marshall, W. (2001) Centrioles take center stage. *Current Biology* 11, R487-R496

88. Lin, R.-T., Takahashi, K., Karjalainen, A., Hoshuyama, T., Wilson, D., Kameda, T., Chan, C.-C., Wen, C.-P., Furuya, S., and Higashi, T. (2007) Ecological association between asbestos-related diseases and historical asbestos consumption: an international analysis. *The lancet* 369, 844-849

89. Matsuda, S., Kawamoto, K., Miyamoto, K., Tsuji, A., and Yuasa, K. (2017) PCTK3/CDK18 regulates cell migration and adhesion by negatively modulating FAK activity. *Scientific Reports* 7, 45545

90. Matsuda, S., Kominato, K., Koide-Yoshida, S., Miyamoto, K., Isshiki, K., Tsuji, A., and Yuasa, K. (2014) PCTAIRE kinase 3/cyclin-dependent kinase 18 is activated through association with cyclin A and/or phosphorylation by protein kinase A. *Journal of Biological Chemistry* 289, 18387-18400

91. Wang, R., Su, C., Wang, X., Fu, Q., Gao, X., Zhang, C., Yang, J., Yang, X., and Wei, M. (2018) Global gene expression analysis combined with a genomics approach for the identification of signal transduction networks involved in postnatal mouse myocardial proliferation and development. *International journal of molecular medicine* 41, 311-321

92. Cao, F., Wagner, R. A., Wilson, K. D., Xie, X., Fu, J.-D., Drukker, M., Lee, A., Li, R. A., Gambhir, S. S., and Weissman, I. L. (2008) Transcriptional and functional profiling of human embryonic stem cell-derived cardiomyocytes. *PloS one* 3, e3474

93. Rual, J.-F., Venkatesan, K., Hao, T., Hirozane-Kishikawa, T., Dricot, A., Li, N., Berriz, G. F., Gibbons, F. D., Dreze, M., and Ayivi-Guedehoussou, N. (2005) Towards a proteome-scale map of the human protein–protein interaction network. *Nature* 437, 1173-1178

94. Greifenberg, A. K., Hönig, D., Pilarova, K., Düster, R., Bartholomeeusen, K., Bösken, C. A., Anand, K., Blazek, D., and Geyer, M. (2016) Structural and functional analysis of the Cdk13/Cyclin K complex. *Cell reports* 14, 320-331

95. Bostwick, B. L., McLean, S., Posey, J. E., Streff, H. E., Gripp, K. W., Blesson, A., Powell-Hamilton, N., Tusi, J., Stevenson, D. A., and Farrelly, E. (2017) Phenotypic and molecular characterisation of CDK13-related congenital heart defects, dysmorphic facial features and intellectual developmental disorders. *Genome medicine* 9, 73

96. Chapelin, C., Duriez, B., Magnino, F., Goossens, M., Escudier, E., and Amselem, S. (1997) Isolation of several human axonemal dynein heavy chain genes: genomic structure of the catalytic site, phylogenetic analysis and chromosomal assignment. *FEBS letters* 412, 325-330

97. Mikolcevic, P., Rainer, J., and Geley, S. (2012) Orphan kinases turn eccentric: a new class of cyclin Y-activated, membrane-targeted CDKs. *Cell Cycle* 11, 3758-3768

98. McQuillen, P. S., Goff, D. A., and Licht, D. J. (2010) Effects of congenital heart disease on brain development. *Progress in pediatric cardiology* 29, 79-85

99. Uhlen, M., Oksvold, P., Fagerberg, L., Lundberg, E., Jonasson, K., Forsberg, M., Zwahlen, M., Kampf, C., Wester, K., and Hober, S. (2010) Towards a knowledge-based human protein atlas. *Nature biotechnology* 28, 1248-1250

100. Larkins, C. E., Aviles, G. D. G., East, M. P., Kahn, R. A., and Caspary, T. (2011) Arl13b regulates ciliogenesis and the dynamic localization of Shh signaling proteins. *Molecular biology of the cell* 22, 4694-4703

101. Fan, Y., Esmail, M. A., Ansley, S. J., Blacque, O. E., Boroevich, K., Ross, A. J., Moore, S. J., Badano, J. L., May-Simera, H., and Compton, D. S. (2004) Mutations in a member of the Ras superfamily of small GTP-binding proteins causes Bardet-Biedl syndrome. *Nature genetics* 36, 989-993

102. Lechtreck, K. F., Van De Weghe, J. C., Harris, J. A., and Liu, P. (2017) Protein transport in growing and steady‐state cilia. *Traffic*

103. Wiens, C. J., Tong, Y., Esmail, M. A., Oh, E., Gerdes, J. M., Wang, J., Tempel, W., Rattner, J. B., Katsanis, N., and Park, H.-W. (2010) Bardet-Biedl syndrome-associated small GTPase ARL6 (BBS3) functions at or near the ciliary gate and modulates Wnt signaling. *Journal of Biological Chemistry* 285, 16218-16230

104. Berbari, N. F., Lewis, J. S., Bishop, G. A., Askwith, C. C., and Mykytyn, K. (2008) Bardet–Biedl syndrome proteins are required for the localization of G protein-coupled receptors to primary cilia. *Proceedings of the National Academy of Sciences* 105, 4242-4246

105. Howard, A. D., McAllister, G., Feighner, S. D., Liu, Q., Nargund, R. P., Van der Ploeg, L. H., and Patchett, A. A. (2001) Orphan G-protein-coupled receptors and natural ligand discovery. *Trends in pharmacological sciences* 22, 132-140

106. Loktev, A. V., and Jackson, P. K. (2013) Neuropeptide Y family receptors traffic via the Bardet-Biedl syndrome pathway to signal in neuronal primary cilia. *Cell reports* 5, 1316-1329

107. Seo, S., Baye, L. M., Schulz, N. P., Beck, J. S., Zhang, Q., Slusarski, D. C., and Sheffield, V. C. (2010) BBS6, BBS10, and BBS12 form a complex with CCT/TRiC family chaperonins and mediate BBSome assembly. *Proceedings of the National Academy of Sciences* 107, 1488-1493

108. Abd-El-Barr, M. M., Sykoudis, K., Andrabi, S., Eichers, E. R., Pennesi, M. E., Tan, P. L., Wilson, J. H., Katsanis, N., Lupski, J. R., and Wu, S. M. (2007) Impaired photoreceptor protein transport and synaptic transmission in a mouse model of Bardet–Biedl syndrome. *Vision research* 47, 3394-3407

109. Ross, A. J., May-Simera, H., Eichers, E. R., Kai, M., Hill, J., Jagger, D. J., Leitch, C. C., Chapple, J. P., Munro, P. M., and Fisher, S. (2005) Disruption of Bardet-Biedl syndrome ciliary proteins perturbs planar cell polarity in vertebrates. *Nature genetics* 37, 1135-1140

1. Contributed equally [↑](#footnote-ref-1)
