# Supplementary figures and images for: Cilia interactome with predicted protein–protein interactions reveals connections to Alzheimer’s disease, aging and other neuropsychiatric processes
Source: Sci Rep. 2020 Sep 24;10:15629. doi: 10.1038/s41598-020-72024-4 (PMC7515907; doi:10.1038/s41598-020-72024-4)

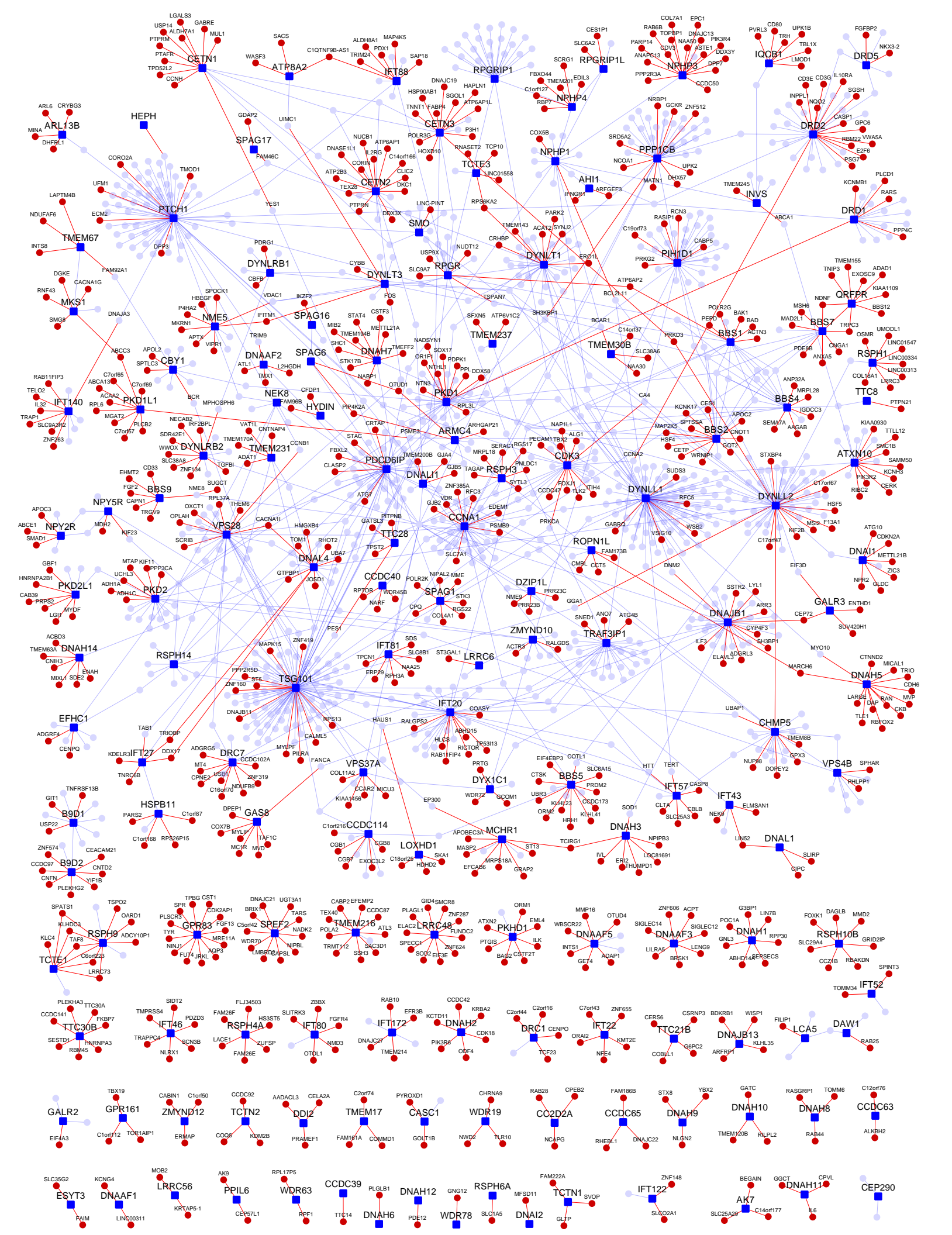

Supplement: Supplementary file 6 — Supplementary Information 6. [file 41598_2020_72024_MOESM6_ESM.pdf]
